# A dual-branch network with brain region-constrained attention for EEG emotion recognition

**DOI:** 10.3389/fnins.2026.1810609

**Published:** 2026-07-07

**Authors:** Chengyu Hua, Hui Cao, Zhaolong Li, Zhiqiang Huang

**Affiliations:** 1 Key Laboratory of Linguistic and Cultural Computing, Ministry of Education, ‘National Languages Information Technology, Northwest Minzu University, Lanzhou, China; 2 Key Laboratory of Minzu Languages and Cultures Intelligent Information Processing, “National Languages Information Technology, Northwest Minzu University, Lanzhou, China

**Keywords:** channel attention calculation, deep learning, electroencephalography (EEG), emotion recognition, multi-domain feature, multi-scale

## Abstract

**Introduction:**

Electroencephalography (EEG)-based emotion recognition provides an objective avenue for affective computing. However, the complexity of EEG signals across temporal, frequency, and spatial domains makes any single dimension inadequate.

**Methods:**

To overcome these limitations, we propose the Brain Region-Constrained Attention Dual-Branch Network (BRAD-Net). This network adopts a parallel Spatio-Temporal and Spectral-Spatial dual-branch architecture to achieve synergistic multi-domain EEG feature learning. Within the spatio-temporal branch, we introduce a novel Brain Region-Constrained Attention mechanism, which strictly confines self-attention computation to channels belonging to the same brain region. This design not only suppresses irrelevant cross-region interference but also incorporates neuroanatomical priors of brain parcellation, thereby enabling effective and interpretable representation learning.

**Results:**

In subject-dependent experiments using 10-fold cross-validation on DEAP and DREAMER datasets, BRAD-Net achieves high accuracies of 97.44%, 97.70%, and 97.97% for valence, arousal, and dominance on DEAP, and 99.66%, 99.78%, and 99.80% on DREAMER, respectively. Leave-one-subject-out validation on DREAMER dataset achieves accuracies of 72.80% and 75.66% for arousal and dominance, respectively. Additionally, the BRAD-Net demonstrates strong cross-paradigm adaptability, achieving 98.21% accuracy on a depression classification dataset.

**Conclusions:**

These findings confirm that integrating neuroanatomical priors into a dual-branch multi-dimensional learning framework effectively extracts robust and interpretable neural representations. BRAD-Net not only advances high-performance EEG emotion recognition but also provides a novel, biologically-constrained design paradigm for developing more interpretable brain-computer interface models. By demonstrating that restricting attention to within-brain-region interactions suffices for accurate emotion recognition, our work offers a new theoretical perspective on the application of brain parcellation knowledge in classification models.

## 1 Introduction

Understanding and objectively identifying emotions is a significant goal in fields ranging from neuroscience and psychology to human-computer interaction. Although questionnaires and scales offer a simple alternative, they are inherently subjective and often biased. Consequently, there is growing interest in finding objective, physiological markers of emotion, with brain activity signals offering a particularly direct window into the neural correlates of our emotional states (Li et al., 2022b). Among various brain imaging techniques, EEG stands out for its non-invasiveness and relative affordability. Neuroscientific studies have consistently shown that different emotional states are associated with distinct, reproducible patterns of brain oscillation across various frequency bands and anatomical regions. This makes EEG an ideal tool for developing computational models of emotion recognition (Xiaomin et al., 2024).

EEG data is rich in information, spanning the time domain (signal waveform), frequency domain (oscillatory power), and space domain (topography across the scalp). Numerous deep learning models have been applied to EEG-based emotion recognition. To address the issue that EEG signals are susceptible to artifacts, Nair et al. (2025) proposed an EEG denoising method that combines a Convolutional Neural Network (CNN) with an LMS adaptive filter, thereby improving EEG signal quality and enhancing the reliability of physiological sleep monitoring. Du et al. (2022) applied self-attention to extract temporal features from EEG for identity recognition. Song et al. (2023) proposed a compact spatio-temporal feature learning model, that uses 1D temporal and spatial convolutional layers to learn low-level local features, which were then fed into a self-attention module to capture global dependencies among different local temporal features. A lightweight model, which consists of a residual network-based feature extractor and a capsule-based classifier, was designed by Fan et al. (2024a) for extracting spatio-temporal features in EEG emotion recognition. For modeling both spatial and temporal characteristics of EEG signals, Jiang et al. (2022) proposed a hybrid convolutional neural network-long short-term memory (CNN-LSTM) framework, where CNNs extract spatial structures and LSTMs capture long-range temporal dependencies in the signals. To incorporate spectral information, a method involving 3D-CNNs processed the two features, Differential Entropy (DE) and Power Spectral Density (PSD), with a dedicated cross-attention module facilitating their fusion (Zhao and Gu, 2024). AB-DPCGRU (Ye et al., 2023) first extracts time-frequency domain features from EEG signals and performs a collaborative analysis with spatial features. On this basis, the model employs a deep convolutional network to mine spatial and frequency domain features from the data, and uses a Gated Recurrent Unit (GRU) to extract temporal domain features, thereby achieving comprehensive modeling of multi-dimensional EEG features through a sequential processing approach. Yang et al. (2026) integrated Simple Graph Convolution for modeling electrode topology with Transformer for capturing long-range channel dependencies, thereby fusing multi-source information to enhance spatial feature extraction. Zhong et al. (2022) employed a regularized graph neural network to model the topological structure of EEG channels, which facilitates the effective learning of intrinsic spatial relationships within EEG signals. However, a critical limitation persists. While emotion is a holistic process involving coordinated dynamics across time, space, and frequency, many existing methods treat these domains in relative isolation or fail to fully exploit their complementary nature (Li et al., 2024; Liu et al., 2023d). This often results in suboptimal feature integration and limited model generalizability.

The spatial distribution of electrode channels holds significant importance for emotion recognition tasks. Many studies have introduced attention mechanisms to address the issue of imbalanced representational capacity across channels. Peng et al. (2023) introduced a channel attention mechanism to assess the role of each EEG channel in emotion analysis, integrating it with a temporal self-attention module for spatio-temporal feature learning. Tao et al. (2023) employed a similar channel-wise attention strategy to weight different channels, combined with CNNs for spatial encoding and an extended self-attention mechanism within recurrent neural networks (RNNs) to refine feature importance based on signal similarity. Besides, for spatial modeling of EEG, graph-based methods have also integrated channel attention mechanisms to leverage the topological relationships among electrodes. A dynamic sparse directed graph convolutional network was developed by Shen et al. (2025) for brain connectivity modeling, where a channel attention mechanism weights electrodes across brain regions. A multi-axis attention mechanism was leveraged by Hou et al. (2024) to aggregate both global and local feature information derived from parallel convolutional branches. A fundamental drawback common to these approaches is their general oversight of established neuroscientific priors regarding functional brain parcellation. By treating all channel interactions as equally plausible, they may capture neurophysiologically implausible dependencies and underutilize the structured spatial information inherent in EEG signals, thereby limiting both interpretability and performance.

Numerous studies have introduced neuroanatomical priors into EEG pattern recognition tasks. Most of these studies adopt a “local brain region + global brain” interaction framework, which quantifies long-range cross-region interactions to adjust the weight allocation of electrode channels, thereby achieving feature optimization. Gao et al. (2026) developed a neuroscience-inspired emotional perception learning system that models local brain-heart dynamic correlations through bilinear fusion and designs a cross-perception mechanism based on dynamic routing to capture global brain-heart bidirectional coupling representations, thereby achieving more robust emotion recognition. Li et al. (2023a) constructed local features based on the rhythmic contributions of different brain regions to identify critical areas, and combined global and region-specific features for fatigue detection. Li et al. (2026) first extracts channel interaction features within each brain region, and then employs global-local fusion and cross-domain adaptive learning to accomplish various EEG pattern recognition tasks. The MEC-MSL method (Gao et al., 2025a) also achieves auditory attention decoding by mining high-dimensional topological representations and modeling both local and global functional connectivity of EEG signals.

This paper innovatively proposes BRAD-Net to address the limitations of the aforementioned studies. Our work introduces two key innovations. First, we design a parallel dual-branch architecture that processes spatio-temporal and spectral-spatial features simultaneously, enabling synergistic and complementary learning across domains to overcome the shortcomings of isolated single-domain modeling.

Second, and most notably, we introduce a novel brain region-constrained attention mechanism within the spatio-temporal branch. Different from previous studies, this paper innovatively adopts a brain region-constrained attention mechanism. By explicitly defining the scope of attention interaction and prohibiting long-range cross-brain-region information exchange, it effectively avoids biologically implausible long-range cross-region interference and reduces the risk of overfitting caused by redundant correlations. Li et al. (2023a) validated that features from a single brain region alone can also enable effective recognition, which aligns well with the design philosophy of this paper, i.e., “mining effective features within brain regions.”

Specifically, our mechanism refines traditional channel-level attention by restricting attention computation to electrodes within predefined functional brain regions. This design aligns the model's learning process more closely with the brain's functional architecture, ensuring physiological plausibility while eliminating redundant cross-region interaction computations, thereby simplifying the model structure and reducing computational overhead. Consequently, it yields more interpretable spatial feature representations that better capture region-specific activation patterns during emotional processing.

In summary, the main contributions of this work are threefold:

(1) We propose a dual-branch architecture that processes spatio-temporal and spectral-spatial features in parallel to achieve complementary multi-domain information integration for emotion classification;
(2) We introduce a neuroscience-inspired attention mechanism into the model. By focusing on attention calculation within brain regions, this enhancement optimizes the feature weighting process and directly elevates classification performance;
(3) We present a paradigm that deeply integrates neuroscientific priors with deep learning. By embedding knowledge of functional brain parcellation as a structural constraint directly into the attention mechanism, we build brain-aligned models that demonstrate enhanced performance by adhering to the organizational principles of neural systems. This knowledge-guided design not only reduces computational redundancy by eliminating unnecessary cross-region computations but also provides a new theoretical perspective on applying brain parcellation knowledge to classification models.

The proposed BRAD-Net achieves state-of-the-art performance on the popular DEAP and DREAMER emotion recognition datasets and demonstrates strong generalization on an depression detection task. These results collectively validate its capacity for learning robust representations from EEG signals and underscore the value of integrating neuroscientific priors into computational models.

## 2 Materials and methods

### 2.1 Model overview

**Figure 1:**
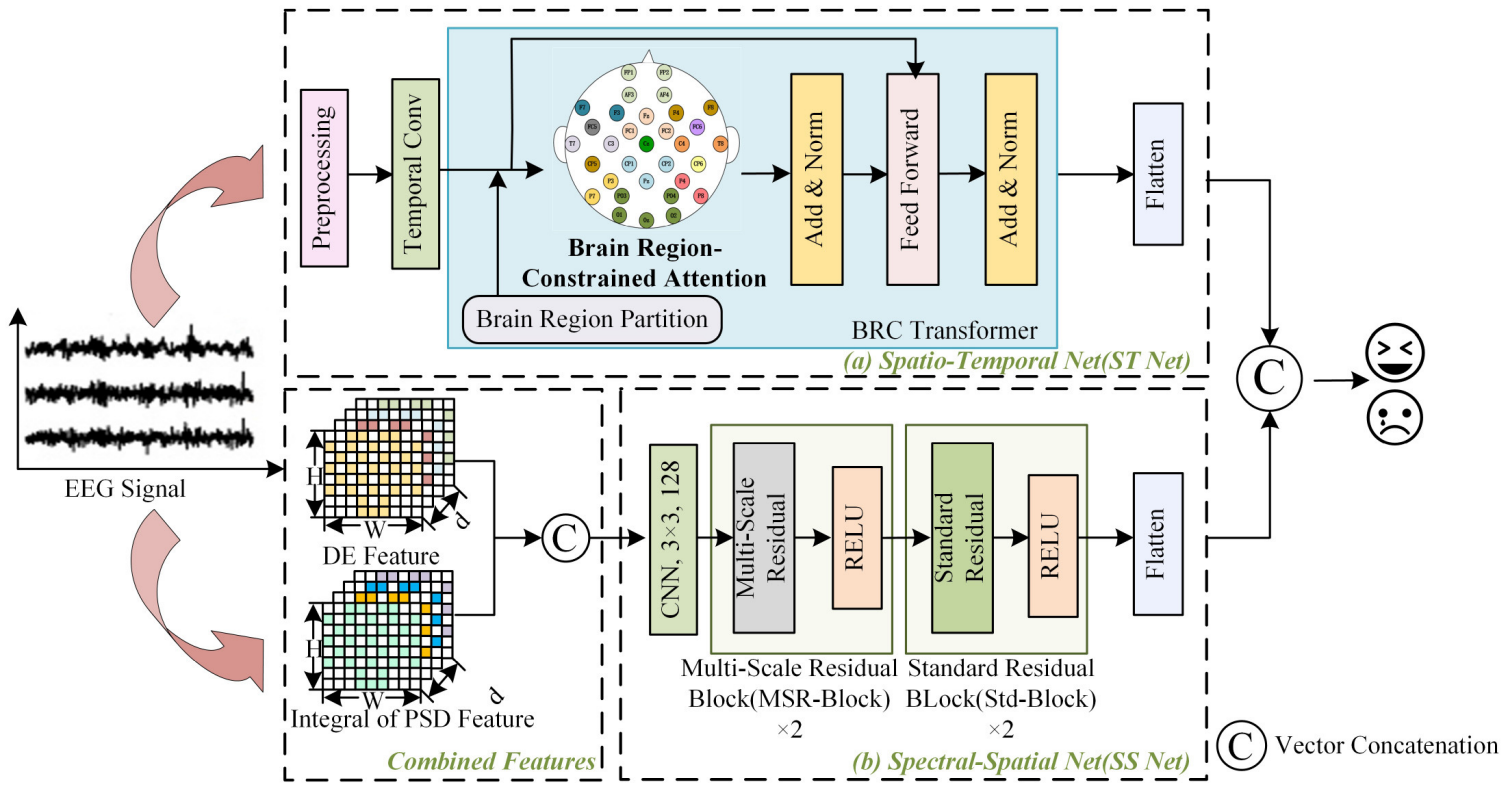
The architecture of the BRAD-Net. BRC transformer: brain region-constrained transformer.

As a physiological signal rich in temporal, spatial, and frequency domain features, EEG provides crucial electrophysiological evidence for emotion recognition research through its complex non-linear information. To fully leverage these multi-domain characteristics, we design BRAD-Net model, the overall architecture of which is depicted in Figure 1. The model consists of a spatio-temporal feature extraction branch and a spectral-spatial feature extraction branch, each dedicated to learning features from different dimensions of the EEG signal.

### 2.2 Data preprocessing and brain region partition

#### 2.2.1 Data preprocessing

First, consistent with previous studies (Jirayucharoensak et al., 2014; Zheng and Lu, 2015), we segment the raw EEG signals into *n* non-overlapping segments of *T* s duration to augment the dataset. Each segment is assigned the original trial's label. A single time segment is represented as $x_{i}\in {\mathbb{R}}^{c\times t}\left(i=1,2\ldots n\right)$, where *c* denotes the number of electrode channels, and *t* = *T*×*f*_*s*_ represents the number of samples within the *T* s window given a sampling rate of *f*_*s*_. All segments *x*_*i*_(*i* = 1, 2…*n*) collectively form a three-dimensional input matrix *X*, where *X*∈ℝ^*n*×*c*×*t*^, which serves as the input to the spatio-temporal branch.

**Figure 2:**
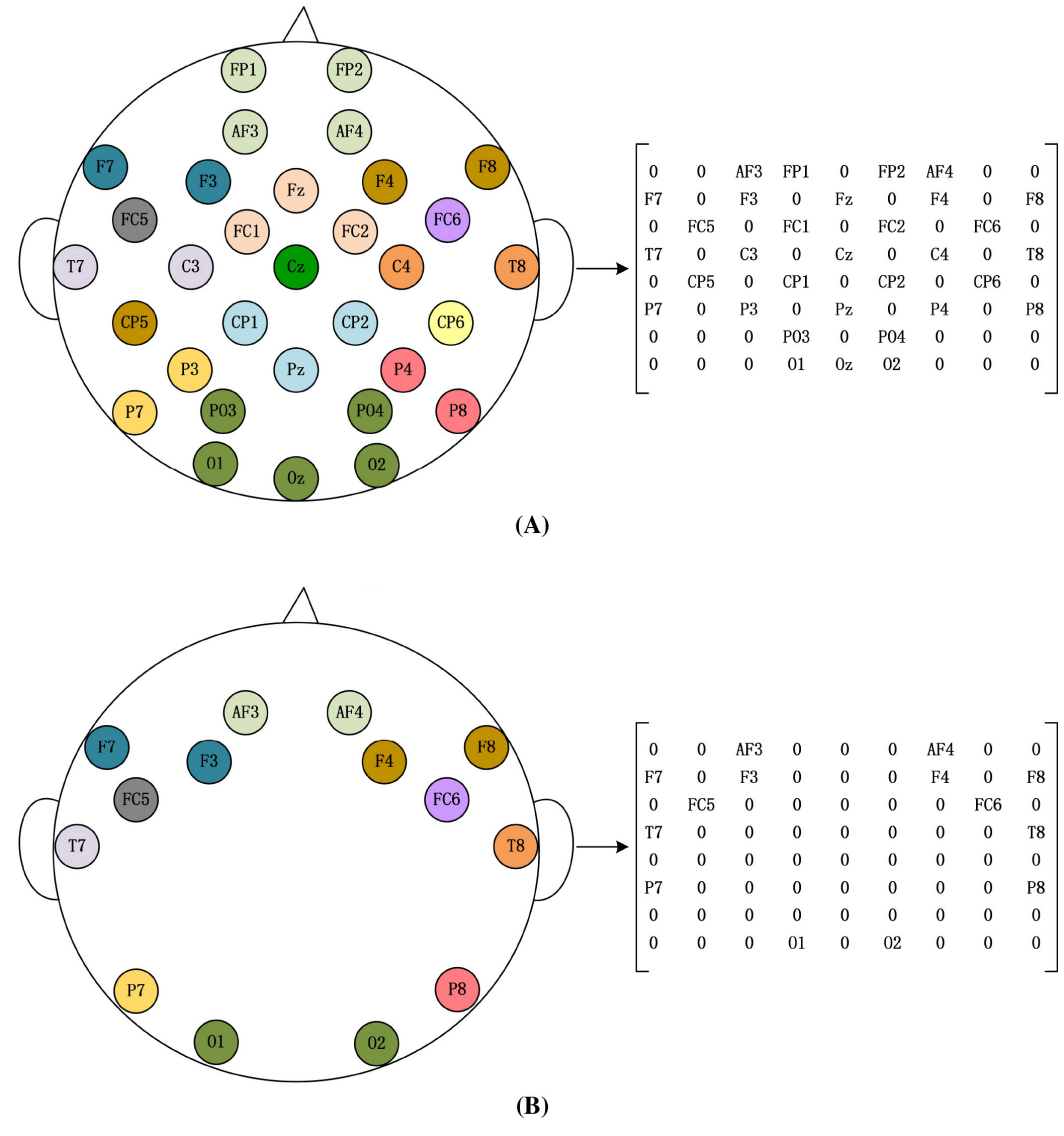
Schematic diagrams of 2D EEG feature matrix construction. **(A)** Mapping of 32 EEG channels from the DEAP dataset onto an 8 × 9 matrix. **(B)** Mapping of 14 EEG channels from the DREAMER dataset onto an 8 × 9 matrix.

Subsequently, for each segment, we employ a Butterworth filter to decompose it into four standard frequency bands: θ (4–8 Hz), α (8–14 Hz), β (14–31 Hz), and γ (31–51 Hz). Within the *T* s window, we calculate the DE feature $y_{1}\in {\mathbb{R}}^{c\times d}$ and the PSD feature. The PSD is then integrated over the window to yield the feature $y_{2}\in {\mathbb{R}}^{c\times d}$, where *d* = 4 corresponds to the number of frequency bands. Following the approach of prior work (Rudakov et al., 2021; Shen et al., 2020) and based on the electrode layout of the international 10-20 system, these features are mapped onto a 2D matrix to form a spatial distribution representation. The specific mapping schemes for the DEAP and DREAMER datasets are illustrated in Figure 2, respectively. Finally, the DE and integrated PSD features from the four frequency bands are stacked along the channel dimension, resulting in a combined feature $y_{j}\in {\mathbb{R}}^{2d\times h\times w}\left(j=1,2,\ldots ,n\right)$. Here, *w* and *h* are the width and height of the 2D map, respectively. All time-frequency features *y*_*j*_(*j* = 1, 2, …, *n*) form the matrix **Y**, where **Y**∈ℝ^*n*×2*d*×*h*×*w*^, which serves as the input to the spectral-spatial branch. In this paper, the window length T is uniformly set to 1, and the 2D map dimensions are *h* = 8 and *w* = 9.

#### 2.2.2 Brain region partitioning

**Figure 3:**
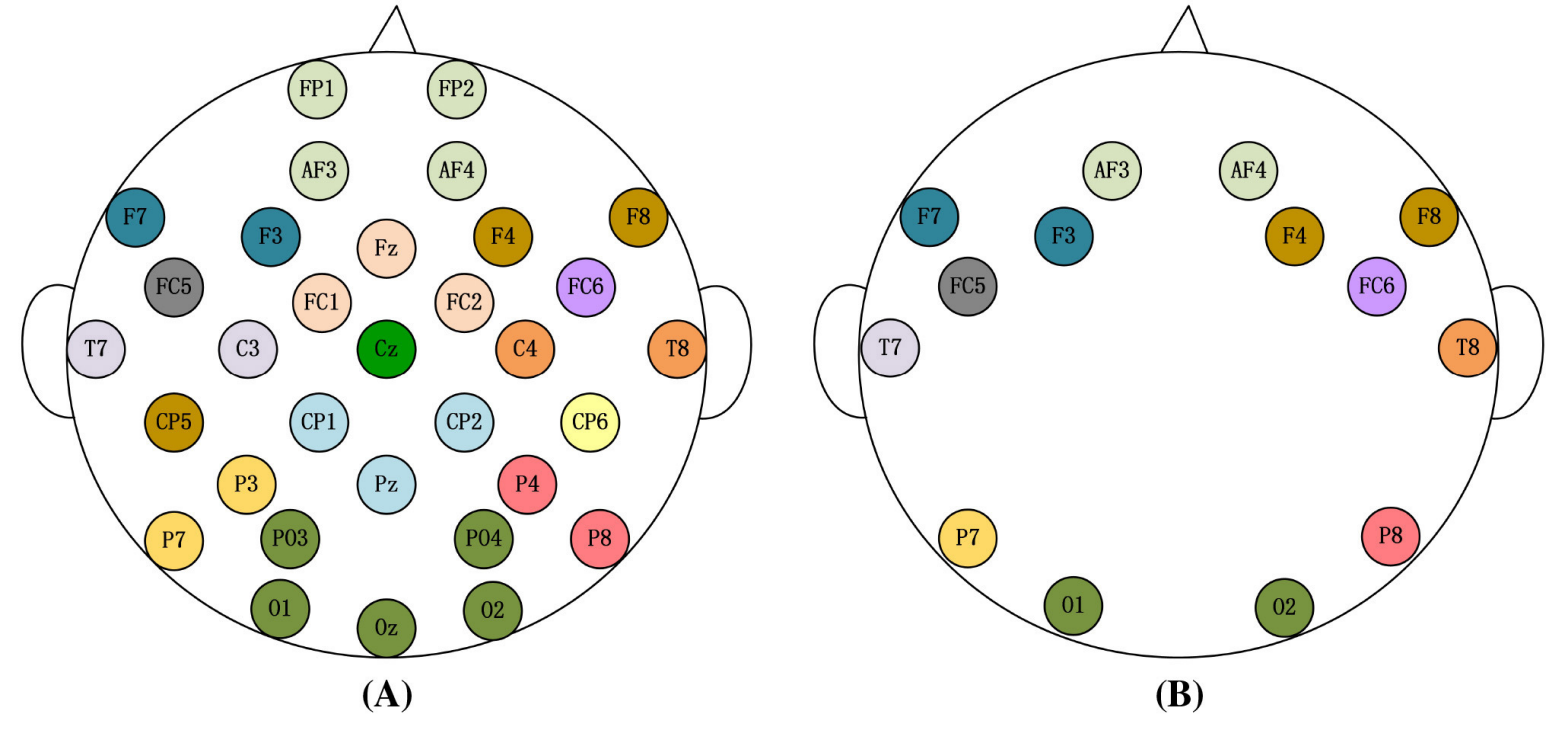
Neurologically-informed brain region parcellation for electrode grouping. Channels of the same color belong to the same functional brain region. **(A)** Neurologically-oriented brain region partitions for the DEAP datasets. **(B)** Neurologically-oriented brain region partitions for the DREAMER datasets.

Neuroscience research indicates that the human brain is organized into specific clusters driven by mechanisms such as physical connectivity, functional synergy, and information flow (Rubinov and Sporns, 2010; Yi et al., 2023). These local brain regions reveal functional patterns of brain activity and provide an operational framework, thereby establishing a theoretical basis for the partitioning of EEG channels into functional groups. Following the optimal brain cluster partitioning explored in Yi et al. (2023); Ye et al. (2022), this paper groups the electrodes of the DEAP and DREAMER datasets based on neurologically-oriented parcellation principles to represent distinct functional brain clusters. Figure 3 show the brain region partitions for the DEAP and DREAMER datasets, respectively, where electrodes marked with the same color belong to the same brain region.

In contrast to the 32 channels employed in the DEAP dataset, only 14 electrode channels are used for data acquisition in the DREAMER dataset. Consequently, the DREAMER dataset omits nine electrodes (FP1, FP2, C3, C4, P3, P4, PO3, PO4, Oz) that are included in DEAP, leading to differences in the corresponding six functional brain regions between the two datasets. Furthermore, nine other electrodes (FC1, FC2, Fz, Cz, CP1, CP2, Pz, CP5, CP6) corresponding to five other brain regions are not utilized in the DREAMER dataset. Based on this, we ultimately partition the 32 channels of the DEAP dataset into 15 brain regions and the 14 channels of the DREAMER dataset into 10 brain regions.

### 2.3 Spatio-temporal feature extraction module

As a bioelectrical signal with distinct spatial topological properties, EEG signals have electrode channel spatial locations closely related to the distribution of functional brain areas. These spatial features hold significant discriminative value for emotion recognition tasks. To capture the spatio-temporal interaction information in EEG signals and the specific weights of different electrode channels, the spatio-temporal net in this paper adopts a hybrid CNN + transformer architecture for temporal feature extraction and channel attention computation, enabling the modeling of spatio-temporal information in EEG signals.

In the temporal information aggregation module, convolutional operations are employed for learning temporal information. Specifically, given an input sample $x_{i}\in {\mathbb{R}}^{C\times T}$, where *C* represents the number of electrode channels. For data segments with a sampling rate of 128 Hz and a window size of 1 s, a one-dimensional convolution with a kernel size of 49 is applied along the time dimension for local temporal feature aggregation. Subsequently, a batch normalization layer is used to standardize the features, avoiding internal covariate shift, gradient vanishing, or explosion, thereby enhancing training stability and improving the model's generalization capability. Following this, the exponential linear unit (ELU) is used as the nonlinear activation function.

**Figure 4:**
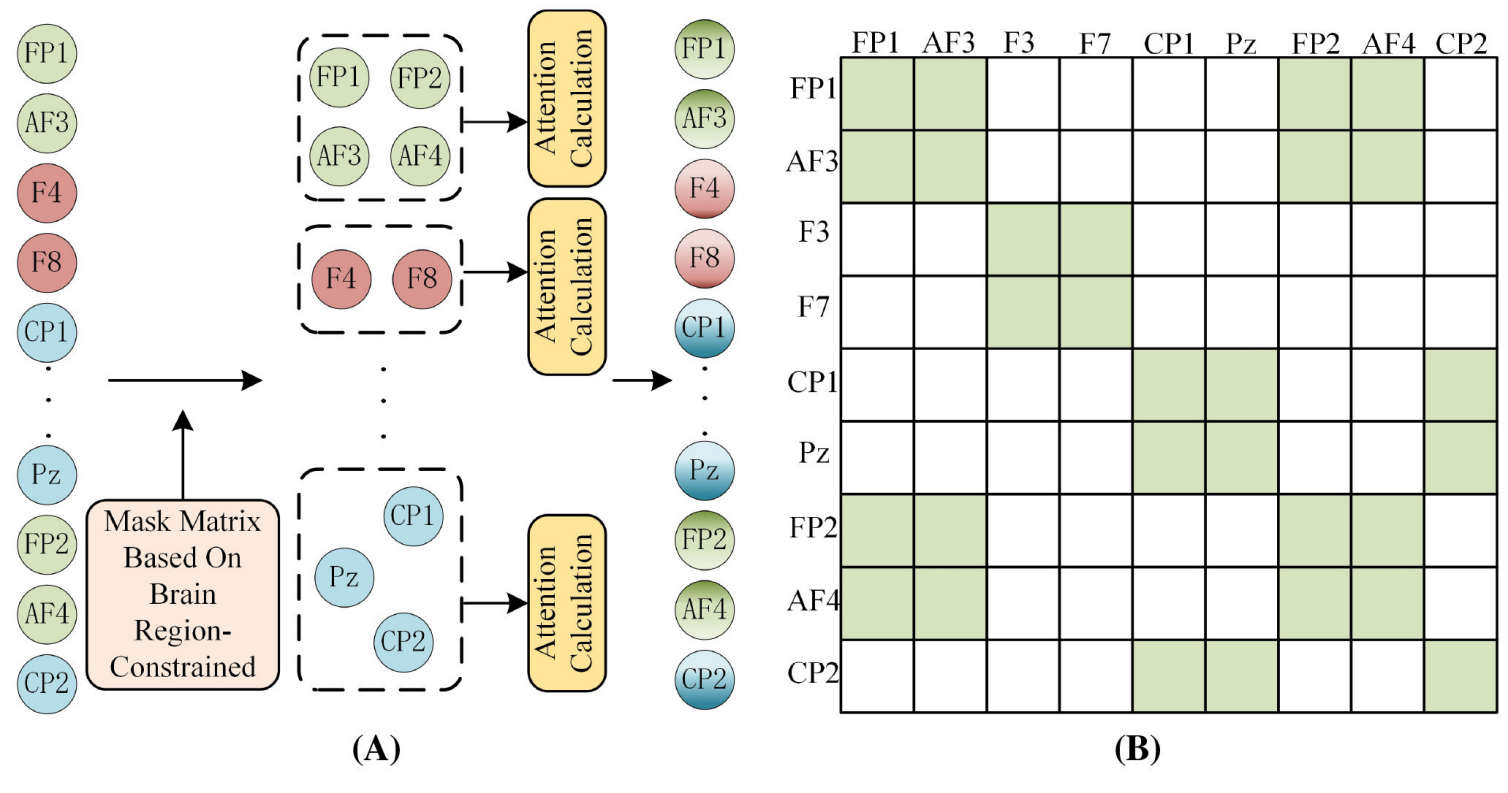
Schematic illustration of the brain region-constrained attention mechanism. **(A)** Workflow for computing attention weights within brain regions. **(B)** Binary constraint mask matrix where green cells (value 0) allow attention, and white cells (value −∞) mask out cross-region interactions.

In the spatial feature modeling stage, this paper innovatively proposes a brain region-constrained attention mechanism. The modified attention computation is illustrated Figure 4. Previous studies using Transformer for channel attention computation often relied solely on positional encoding to represent sequence order, performing undifferentiated global attention calculations across all channels. This approach loses the spatial adjacency of electrodes and the local information of brain regions on the scalp. Different from traditional global attention methods, this approach divides electrode channels into several brain regions based on neuroscientific prior knowledge. Taking a subset of channels from Figure 4 as an example, the colored areas indicate that attention computation is allowed between channels, whereas the masked areas prevent any attention computation. During the multi-head attention computation, a mask matrix *M*∈ℝ^*C*×*C*^ is defined, with its elements defined as Equation 1:


$$\begin{array}{l} M_{i,j}=\{\begin{array}{ll} 0 & \text{if }i,j\text{ are equal or in the same}\\ -\infty & \text{otherwise} \end{array} \end{array}$$ (1)

The brain region-constrained masking strategy modified the attention score matrix as Equation 2:


$$\begin{array}{l} S=\frac{QK^{T}}{\sqrt{d_{k}}}+M \end{array}$$ (2)

When *M*_*i, j*_ = 0, the attention score retains its original computed value, allowing complete dependency relationships to be established within functional groups; when *M*_*i, j*_ = −∞, after the Softmax function transformation, the corresponding *S* approximates 0. Using the mask matrix obscures irrelevant information from other brain regions, allowing dependency computation only within brain regions to exclude spurious correlations from cross-regional interference. The modified attention mechanism is expressed as Equation 3:


$$\begin{array}{l} Attention\left(Q,K,V\right)=Softmax\left(\frac{QK^{T}}{\sqrt{d_{k}}}+M\right)V \end{array}$$ (3)

Since neural activities within the same brain region exhibit high functional consistency, performing attention computation within brain regions better aligns with the physiological characteristics of functional brain partitioning.

### 2.4 Spectral-spatial feature extraction module

The spectral-spatial branch is formed by two multi-scale residual blocks and two standard residual blocks connected sequentially. All convolutional operations are implemented using depthwise separable convolutions, which significantly reduce the model's parameter count by decomposing the standard convolution into a depthwise convolution followed by a pointwise convolution. For an input sample $y_{i}\in {\mathbb{R}}^{2d\times h\times w}$, a feature dimension expansion is first performed along the frequency band dimension, expanding the feature from $y_{i}\in {\mathbb{R}}^{2d\times h\times w}$ to $y_{j}\in {\mathbb{R}}^{128\times h\times w}$. The processed features are then fed into the multi-scale residual blocks for subsequent feature extraction.

**Figure 5:**
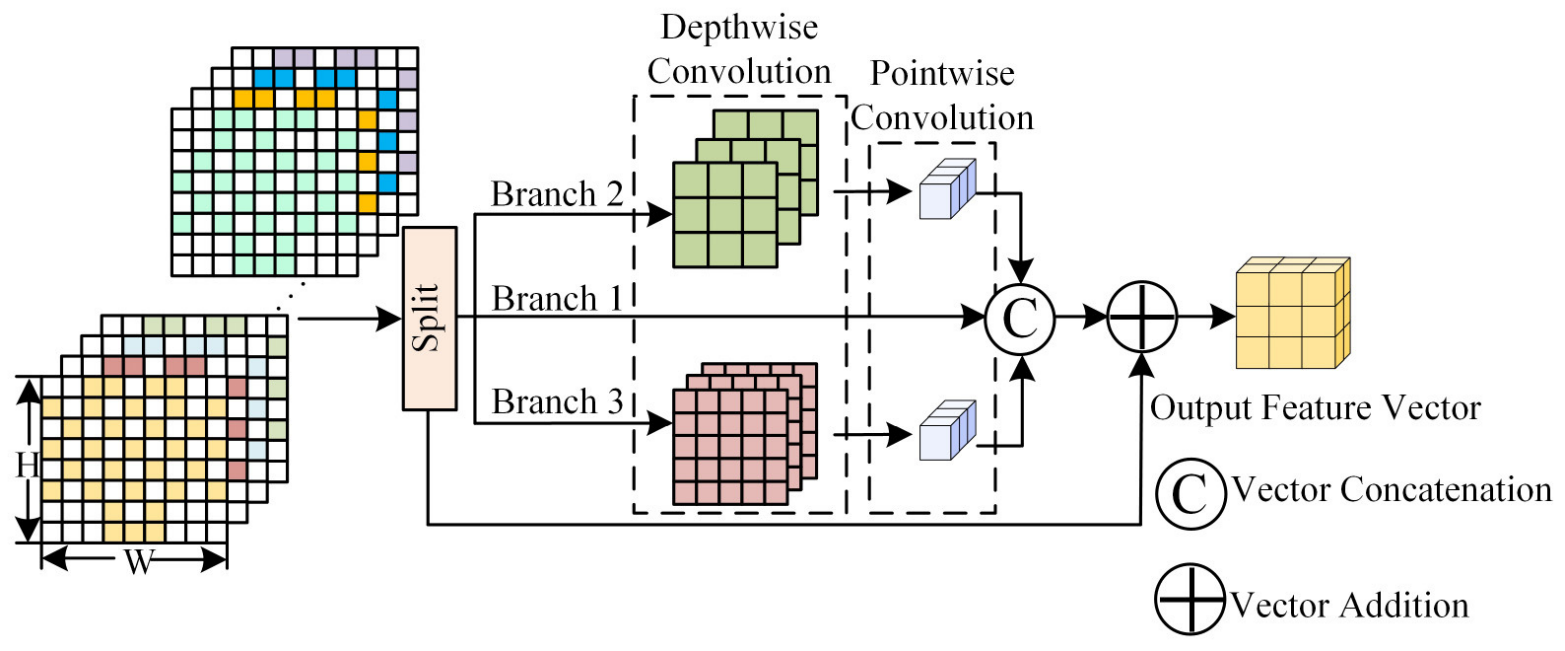
Architecture of the multi-scale residual block.

As illustrated in Figure 5, the multi-scale residual block is designed with a branched, hierarchical fusion architecture, employing a three-branch parallel structure to achieve multi-granularity feature extraction. The input is split into three parts: $y_{j}^{1}\in {\mathbb{R}}^{d_{1}\times h\times w}$, $y_{j}^{2}\in {\mathbb{R}}^{d_{2}\times h\times w}$,$y_{j}^{3}\in {\mathbb{R}}^{d_{3}\times h\times w}$, where *d*_1_+*d*_2_+*d*_3_ = 128. These three parts are processed by the three branches, respectively. Branch 1 is an identity mapping path without any convolution, preserving the original information flow as formulated in Equation 4:


$$\begin{array}{l} B_{1}\left(y_{j}^{1}\right)=y_{j}^{1} \end{array}$$ (4)

Branch 2 utilizes a 3 × 3 convolutional kernel to extract local features with stride=1, padding=1, implemented via depthwise separable convolution as defined in Equation 5:


$$\begin{array}{l} B_{2}\left(y_{j}^{2}\right)=\text{ReLU}\left(\text{BN}\left({\text{Conv}}_{1\times 1}\left({\text{Conv}}_{3\times 3}^{\text{depthwise}}\left(y_{j}^{2}\right)\right)\right)\right) \end{array}$$ (5)

Branch 3 employs a 5 × 5 kernel to capture features with a larger receptive field, with stride=1, padding=2, computed according to Equation 6:


$$\begin{array}{l} B_{3}\left(y_{j}^{3}\right)=\text{ReLU}\left(\text{BN}\left({\text{Conv}}_{1\times 1}\left({\text{Conv}}_{5\times 5}^{\text{depthwise}}\left(y_{j}^{3}\right)\right)\right)\right) \end{array}$$ (6)

The output features from the three branches are concatenated, and the resulting feature map is then combined with the module's original input via a residual connection, formulated as Equation 7:


$$\begin{array}{l} Y_{output}=y_{j}+{\text{Conv}}_{1\times 1}\left(\text{Concat}[B_{1}\left(y_{j}^{1}\right),B_{2}\left(y_{j}^{2}\right),B_{3}\left(y_{j}^{3}\right)]\right) \end{array}$$ (7)

By enabling collaborative feature extraction across heterogeneous receptive fields, the multi-scale residual block enhances the model's capacity to learn representations from various frequency bands while remaining parameter-efficient.

Following the multi-scale residual blocks, two standard residual blocks with a “bottleneck” design are connected. The first residual block employs a 3 × 3 grouped convolution kernel, while the second uses a 5 × 5 grouped convolution kernel. In each bottleneck block, the input dimensionality is first reduced to one-quarter of its original size via a pointwise convolution, followed by grouped convolution for feature extraction, and then another pointwise convolution expands the dimensionality to half of the current input dimension. Specifically, the first bottleneck residual block compresses the 128 dimensional input feature to 64 dimensions, and the second bottleneck residual block further compresses the 64 dimensional feature to 32 dimensions. Consequently, the original 128 dimensional input feature is progressively compressed to 32 dimensions after passing through these two residual blocks.

### 2.5 Fusion module and classifier

The feature vectors from both branches, respectively carrying rich spatio-temporal and spectral-spatial information, are flattened and then concatenated into a combined representation that fully preserves the rich information from temporal waveforms, spectral power, and spatial topologies.

Finally, we employ a fully connected layer as the classifier. This layer maps the fused multi-domain feature vector onto the label space for the classification task. The final output of the network was an N-dimensional vector, where *N* represents the number of emotion categories for the EEG classification task. The entire model is optimized using the cross-entropy loss function.

## 3 Results

### 3.1 Emotion EEG datasets

The DEAP dataset (Koelstra et al., 2012) is a multimodal emotion database that includes EEG signals and other peripheral physiological signals from 32 participants. To induce emotional reactions in the subjects, a total of 40 video clips, each lasting 1 min, were presented during the experiment. After watching each video, participants rated their emotional state on the Self-Assessment Manikin scale across three dimensions: arousal, valence, and dominance, on a discrete scale from 1 to 9. This paper utilizes the officially preprocessed version of the DEAP dataset, where the signals were down-sampled to a 128 Hz sampling rate, a band-pass filter was applied to extract data in the 4.0–45.0 Hz frequency range, and blind source separation was employed for artifact removal. Since the label scores in the DEAP dataset range from 1 to 9, a threshold of 5 was applied to the scores for arousal, valence, and dominance to categorize the signals into binary classes (high and low) for each dimension.

The DREAMER dataset (Katsigiannis and Ramzan, 2018) was constructed by collecting EEG signals from 23 subjects, who were instructed to watch 18 film clips while their signals were recorded through 14 channels. All EEG signals were recorded at a 128 Hz sampling rate, filtered using a band-pass Hamming windowed since linear-phase FIR filter, and subsequently cleansed of artifacts using the Source Subspace Reconstruction method. The duration of each film clip ranged from 65 to 393 s, which was sufficient to evoke an emotional state. After watching each clip, every subject rated their corresponding emotional responses on a 1–5 scale. Based on this scoring range, a threshold of 3 was used to convert the continuous ratings for arousal, valence, and dominance into discrete binary labels (high/low).

### 3.2 Experimental details

Our model is implemented using the PyTorch framework, and all experiments are conducted on an NVIDIA A800 GPU. Following the methodology described in Yang et al. (2018), baseline signals are removed from all time-series data as well as the computed DE features and integrated PSD features. This preprocessing step helps eliminate low-frequency drifts and baseline shifts, ensuring that the subsequent feature extraction focuses on task-relevant neural activity.

A simple data augmentation strategy involving random cropping and temporal shuffling is applied during the training phase. Specifically, we randomly select 54 starting points without exceeding the sequence boundaries within the data of the same trial. At each starting point, a contiguous segment of 10 data points is cropped. These 540 data points are then downsampled to form complete samples. These samples are appended to the training set to expand the data size. We perform shuffling between cropped segments while keeping the temporal order within each segment unchanged. This ensures that local physiological features, such as the energy distribution across specific frequency bands and local waveform morphology, are fully preserved, while the combination of cropped segments enriches the training data. The training settings are summarized in Table 1.

**Table 1:** Experimental parameter settings.

Parameter	DEAP		DREAMER	
	Subject-dependent	Subject-independent	Subject-dependent	Subject-independent
Optimizer	Adam (Kingma and Ba, 2014)	Adam (Kingma and Ba, 2014)	Adam (Kingma and Ba, 2014)	Adam (Kingma and Ba, 2014)
Epochs	50	125	50	125
Batch size	128	512	128	128
Learning rate	0.001	0.001	0.001	0.001
Weight decay	0.003	0.003	0.003	0.003

To ensure the stability and reproducibility of the experimental results, this study adopts a comprehensive randomness control strategy that fixes all potential sources of randomness. At the system level, environment variables are set to enforce deterministic CUDA convolution operations and to fix Python hash randomization. At the framework level, PyTorch's deterministic mode is enabled to ensure that convolution operators select the same algorithm implementation in each run. At the data level, fixed random seeds are set for NumPy and Python's built-in random number generators, and independent random states are assigned to each subprocess of the DataLoader. The above strategies guarantee that all experimental results can be accurately reproduced under identical hardware and software environments.

### 3.3 Subject-dependent experimental results

The performance of the proposed method was evaluated using 10-fold cross-validation in subject-dependent experiments. All trial data from the same subject were shuffled, and data partitioning was performed at the window level. Since the 1 s time windows are non-overlapping and exhibit no significant temporal dependence, and the DataLoader performs sample shuffling, no data leakage occurs. The average performance across the 10-fold validation process was computed as the final result for each individual subject. The mean accuracy across all subjects was subsequently reported as the overall performance metric. To thoroughly demonstrate the effectiveness of our approach, we conducted extensive experiments on both datasets and compared the results with three deep learning baseline models including a two-layer CNN, LSTM (Graves, 2012), and Transformer (Vaswani et al., 2017), in addition to state-of-the-art methods. For models that reported subject-level accuracy in their original papers, we performed paired t-tests. The results demonstrate that the differences are highly statistically significant for the vast majority of comparisons, further confirming that the performance advantages of our model are not due to chance.

We employed our proposed classification model to perform binary classification on the arousal, valence, and dominance dimensions for all 32 subjects in the DEAP dataset and 23 subjects in the DREAMER dataset. The accuracy, F1-score, recall, and precision were calculated for each of the three dimensions, with detailed results presented in Table 2. The mean values and standard deviations indicate well-balanced and excellent performance across both datasets. Comparative results of classification accuracy between our model and other existing approaches are provided in Tables 3, 4. The results demonstrate that our model achieves significant improvement in classification accuracy across all three dimensions on both datasets.

**Table 2:** Performance evaluation using subject-dependent 10-fold cross-validation across three emotional dimensions on DEAP and DREAMER.

Dataset	Indicator	Performance (mean ±std, %)		
		Valence	Arousal	Dominance
DEAP	Accuracy	97.44 ± 1.19	97.70 ± 1.13	97.97 ± 1.14
	F1-score	97.65 ± 1.15	97.82 ± 1.45	98.18 ± 1.07
	Recall	97.70 ± 1.28	97.88 ± 1.75	97.36 ± 1.18
	Precision	97.63 ± 1.13	97.79 ± 1.26	98.03 ± 1.06
DREAMER	Accuracy	99.66 ± 0.22	99.78 ± 0.14	99.80 ± 0.16
	F1-score	99.69 ± 0.23	99.84 ± 0.12	99.85 ± 0.15
	Recall	99.72 ± 0.27	99.88 ± 0.13	99.90 ± 0.17
	Precision	99.68 ± 0.23	99.81 ± 0.12	99.80 ± 0.18

**Table 3:** Classification accuracy comparison for subject-dependent experiments using 10-fold cross-validation on the DEAP dataset.

Model	Valence	Arousal	Dominance
Two-layer CNN	96.78	97.06	97.03
LSTM (Graves, 2012)	83.94	85.50	85.89
Transformer (Vaswani et al., 2017)	94.82	94.91	94.93
TR&CA (Peng et al., 2023)	95.18	95.58	95.78
DAMGCN (Chen et al., 2024)	96.96	97.17	97.50
Bi-AAN (Zhong et al., 2023)	96.96	96.63	-
STLGCNN (Xu et al., 2024)	93.81	94.16	93.88
AP-CapsNet (Liu et al., 2023a)	93.89	95.04	95.08
STC-CNN (Li et al., 2023b)	96.79	96.89	-
Caps-EEGNet (Chen et al., 2023)	96.67	96.75	96.64
CNN-BiLSTM-CS (Zhang et al., 2025)	94.22	92.16	-
GLFA-Net (Liu et al., 2023b)	94.53	94.91	95.35
HA-CapsNet (Chen et al., 2025)	97.20	97.40	97.60
DSSA Net (Liu et al., 2025)	94.97	94.73	-
MTSL-TimesNet (Li et al., 2025)	95.92	96.30	-
**BRAD-Net (ours)**	**97.44**	**97.70**	**97.97**

The bold values indicate the best results.

**Table 4:** Classification accuracy comparison for subject-dependent experiments using 10-fold cross-validation on the DREAMER dataset.

Model	Valence	Arousal	Dominance
Two-layer CNN	98.26	98.89	98.72
LSTM (Graves, 2012)	80.43	85.99	88.29
Transformer (Vaswani et al., 2017)	86.17	90.42	91.64
LResCapsule (Fan et al., 2024a)	95.77	95.15	95.59
Bi-AAN (Zhong et al., 2023)	92.68	92.95	-
Caps-EEGNet (Chen et al., 2023)	91.12	92.60	93.74
GLFA-Net (Liu et al., 2023b)	94.82	94.57	94.51
ICaps-ResLSTM (Fan et al., 2024b)	94.97	94.97	94.96
MTCA-CapsNet (Li et al., 2022a)	95.54	94.96	95.52
MLF-CapsNet (Liu et al., 2020)	94.59	95.26	95.13
FP-CapsNet (Liu et al., 2023c)	95.86	95.48	95.86
FC-TFS-CGRU (Wu et al., 2024)	98.63	98.7	98.71
RGNet-GCN (Fan et al., 2024c)	99.17	99.06	99.23
PconvCapsNet (Lin et al., 2026)	96.49	97.20	97.04
HA-CapsNet (Chen et al., 2025)	95.80	96.10	96.30
4D-MRSIMNET (Huang and Deng, 2026)	99.60	99.46	-
MTSL-TimesNet (Li et al., 2025)	90.66	90.76	-
**BRAD-Net (ours)**	**99.66**	**99.78**	**99.80**

The bold values indicate the best results.

To validate the effectiveness of our multi-dimensional feature fusion strategy, we systematically compared BRAD-Net with several representative multi-domain feature extraction models.

For capsule network-based optimization models, compared to HA-CapsNet (Chen et al., 2025), which integrates channel attention and frequency band attention, BRAD-Net achieves improvements of 0.24%, 0.30%, and 0.37% on the valence, arousal, and dominance dimensions, respectively; on the DREAMER dataset, the improvements are 3.68%, 3.68%, and 3.50%, respectively. Compared to Caps-EEGNet (Chen et al., 2023) combined with EEGNet and channel selection, our model achieves improvements of 0.77%, 0.95%, and 1.33% on the DEAP dataset, and improvements of 8.54%, 7.18%, and 6.06% on the DREAMER dataset. Compared to AP-CapsNet (Liu et al., 2023a), which employs pre-training and coordinate attention, our model achieves improvements of 3.55%, 2.66%, and 2.89% on the three dimensions of the DEAP dataset. Compared to FP-CapsNet (Liu et al., 2023c), a lightweight binarized capsule network, our model surpasses it by 3.80%, 3.90%, and 3.94% on the DREAMER dataset. Compared to MLF-CapsNet (Liu et al., 2020) with multi-level feature guidance, our model outperforms it by 5.07%, 4.52%, and 4.67% on the three dimensions of DREAMER. Compared to ICaps-ResLSTM (Fan et al., 2024b), which integrates an improved capsule network with residual connections, our model outperforms it by 4.69%, 4.81%, and 4.84% on DREAMER.

For CNN and graph convolution-based classification methods, compared to STC-CNN (Li et al., 2023b), which uses convolutional kernels of different sizes to extract short-range and long-range spatial features, our model outperforms it by 0.65% and 0.81% on the valence and arousal dimensions of the DEAP dataset. Compared to STLGCNN (Xu et al., 2024), which first constructs graph structures and then integrates temporal dynamics via LSTM, our model achieves improvements of 3.63%, 3.54%, and 4.09% on the three dimensions of DEAP.

For hybrid network methods, compared to CNN-BiLSTM-CS (Zhang et al., 2025), which combines CNN and bidirectional LSTM with joint loss supervision, our model achieves improvements of 3.22% and 5.54% on two dimensions of DEAP. Compared to FC-TFS-CGRU (Wu et al., 2024), which constructs frequency-space matrices and employs CNN-GRU, BRAD-Net surpasses it by 1.03%, 1.08%, and 1.09% on the three dimensions of the DREAMER dataset. Compared to 4D-MRSIMNet (Huang and Deng, 2026), which constructs a 4D spatio-temporal-frequency representation and employs multi-resolution convolution, our model outperforms it by 0.06% and 0.32% on two dimensions of DREAMER.

For attention mechanism and Transformer-based methods, compared to TR&CA (Peng et al., 2023), which jointly utilizes temporal and spatial attention, BRAD-Net achieves improvements of 2.26%, 2.12%, and 2.19% on the three dimensions of the DEAP dataset (*p* < 0.0001, *t* = 9.18). Compared to DSSA Net (Liu et al., 2025), which employs parallel position, spectrum, and time attention modules, our model achieves improvements of 2.47% and 2.97% on the valence and arousal dimensions of the DEAP dataset.

For self-supervised and multi-task learning methods, compared to MTSL-TimesNet (Li et al., 2025) based on multi-task self-supervised learning, our model achieves improvements of 1.52% and 1.40% on the valence and arousal dimensions of the DEAP dataset. Compared to MTCA-CapsNet (Li et al., 2022a), which combines multi-task learning with capsule networks, our model achieves improvements of 4.12%, 4.82%, and 4.28% on the three dimensions of the DREAMER dataset. Compared to MTSL-ERM, which employs dual self-supervised tasks, BRAD-Net outperforms it by 9.00% and 9.02% on two dimensions of DREAMER.

The above comparison methods cover mainstream technical approaches, including capsule networks, graph convolutional networks, recurrent networks, hybrid networks, attention mechanisms, and self-supervised learning, all of which perform feature extraction from spatio-temporal-frequency multi-dimensions. BRAD-Net demonstrates consistent performance advantages across all types of comparisons, fully validating the effectiveness of the proposed dual-branch parallel architecture in integrating temporal dynamics with spectral-spatial features and demonstrating the superiority of the multi-dimensional feature fusion framework.

DAMGCN (Chen et al., 2024) constructs an adjacency matrix based on the three-dimensional spatial distances between electrode channels, aggregates local and global brain region information through graph convolution, and assigns weights using a dual attention mechanism. BRAD-Net surpasses DAMGCN by 0.48%, 0.53%, and 0.47% on the three dimensions of the DEAP dataset.

Bi-AAN (Zhong et al., 2023) similarly integrates neuroscientific knowledge with deep learning models, combining the Transformer architecture with the hemispheric lateralization characteristics of emotional brain responses. BRAD-Net outperforms Bi-AAN by 0.48% and 1.07% on two dimensions of the DEAP dataset, and by 6.98% and 6.83% on two dimensions of the DREAMER dataset. On the valence dimension of DEAP, the statistical significance of the difference is relatively low (*p* = 0.02>0.05, *t* = 2.50), while on the arousal dimension, the difference is highly significant (*p* < 0.0001, *t* = 4.78). This phenomenon may suggest that hemispheric lateralization plays a more important role in valence recognition, whereas arousal recognition may depend more on other brain region mechanisms.

GLFA-Net (Liu et al., 2023b) fuses graph convolutional networks (global features) with convolutional networks (local features), overcoming the limitation of single graph convolution that weakens single-channel deep feature extraction, thereby achieving global-to-local feature aggregation. Compared to GLFA-Net, our model achieves improvements of 2.91%, 2.79%, and 1.72% on the three dimensions of DEAP (valence: *p* < 0.0001, *t* = 10.76; arousal: *p* < 0.0001, *t* = 10.06; dominance: *p* < 0.0001, *t* = 8.20), and improvements of 4.84%, 5.21%, and 5.29% on the DREAMER dataset (valence: *p* < 0.0001, *t* = 6.82; arousal: *p* < 0.0001, *t* = 7.49; dominance: *p* < 0.0001, *t* = 8.48).

Compared to LResCapsule (Fan et al., 2024a), which combines a lightweight residual network with a capsule network to explicitly model local-global spatial relationships, BRAD-Net achieves improvements of 3.89%, 4.63%, and 4.21% on the three dimensions of the DREAMER dataset. RGNet-GCN (Fan et al., 2024c) extracts within-brain-region features through a region encoder and learns inter-brain-region interactions using a dynamic adjacency matrix. Our model surpasses it by 0.49%, 0.72%, and 0.57% on the three dimensions of the DREAMER dataset.

The above methods incorporate neuroscientific knowledge to improve recognition performance by leveraging left-right brain asymmetry or combining local and global information, while retaining information interaction channels between distant brain regions. In contrast, BRAD-Net adopts a more parsimonious computational strategy, focusing solely on within-brain-region information interaction and completely prohibiting cross-brain-region attention computation. BRAD-Net demonstrates competitive performance across all comparisons, validating the rationality of its design and demonstrating that long-range cross-brain-region interactions contain a certain degree of redundant information in EEG-based emotion recognition tasks, and that focusing on local information coupling within brain regions is sufficient to learn robust and discriminative emotion-related features.

### 3.4 Subject-independent experimental results

To provide a more comprehensive evaluation of the model's capabilities, subject-independent experiments were additionally performed in challenging cross-subject scenarios. This paradigm tests the model's adaptability to EEG signals from new, unseen subjects. The subject-independent experiments employed the same PyTorch framework, hardware setup, and data preprocessing procedures as the subject-dependent experiments. We adopted leave-one-subject-out(LOSO) cross-validation, where one subject was sequentially selected as the test set while the remaining subjects constituted the training set.

We performed these experiments on the valence dimension of the DEAP dataset and the arousal and dominance dimensions of the DREAMER dataset. The results demonstrate that our model maintains exceptional classification performance even when subject-specific biases are excluded, achieving competitive accuracy compared to current methods, as detailed in Table 5. Specifically, on the valence dimension of the DEAP dataset, BRAD-Net achieves a classification accuracy of 63.36%. Although this result is lower than the best-performing PconvCapsNet (Lin et al., 2026) (74.01%), it still significantly outperforms classical baseline models such as two-layer CNN (53.41%) and LSTM (Graves, 2012) (61.97%), placing it at an above-average level. On the dominance and arousal dimensions of the DREAMER dataset, our model achieves the best performance among all compared methods. This strongly validates that the emotional representations learned by our method are universal, capturing stable neural patterns across subjects rather than overfitting to individual idiosyncrasies, thereby highlighting its significant advantage in addressing the fundamental challenge of cross-subject emotion recognition.

**Table 5:** Classification accuracy comparison for subject-independent experiments using LOSO cross-validation on the DREAMER dataset and DEAP dataset.

	DREAMER		DEAP
Model	Arousal	Dominance	Valence
Two-layer CNN	69.51	73.28	53.41
LSTM (Graves, 2012)	71.77	74.49	61.97
LResCapsule (Fan et al., 2024a)	63.59	65.01	61.52
DuMoNet (Miao et al., 2025)	71.60	-	68.10
STRFLNet (Hu et al., 2025)	65.76	-	-
Xu et al. (Xu et al., 2025)	66.22	-	61.16
TARDGCN (Li et al., 2023c)	67.98	70.28	57.73
Maheshwari et al. Maheshwari et al. (2021)	51.23	51.69	-
Priyasad et al. Priyasad et al. (2022)	63.71	61.81	69.43
AC-DCL (Gao et al., 2025b)	72.31	73.23	-
PconvCapsNet (Lin et al., 2026)	69.32	70.13	**74.01**
**BRAD-Net (ours)**	**72.80**	**75.66**	63.36

The bold values indicate the best results.

Furthermore, by adopting a streamlined within-brain-region attention strategy and a lightweight multi-scale convolutional module, BRAD-Net achieves a reduction in the number of parameters of approximately 92% compared to PconvCapsNet (Lin et al., 2026), approximately 97% compared to Priyasad (Priyasad et al., 2022), approximately 5.5% of LResCapsule (Fan et al., 2024a), and less than 11% of STRFLNet (Hu et al., 2025) (see Section 4.5 for detailed statistics). This advantage makes it more suitable for deployment on resource-constrained edge devices or wearable systems.

Comparison of the two experimental paradigms reveals that cross-subject classification accuracy is significantly lower than that of subject-dependent experiments. This phenomenon fundamentally stems from the inherent challenges of cross-subject generalization in EEG signals. Significant individual differences exist among subjects of different genders and ages in terms of psychological states and physiological structures-such as variations in head shape and attention level-leading to shifts in the data distribution of EEG signals across individuals. Furthermore, there are considerable differences in long-term lifestyle habits and emotional-cognitive responses among different subjects. The coupling of these factors further exacerbates the difficulty of cross-subject pattern recognition. Consequently, neural activities evoked by the same emotional stimulus often exhibit high variability across different subjects, making it difficult for models to learn a unified and generalizable emotional representation from limited training samples.

## 4 Discussion

### 4.1 Confusion matrix

**Figure 6:**
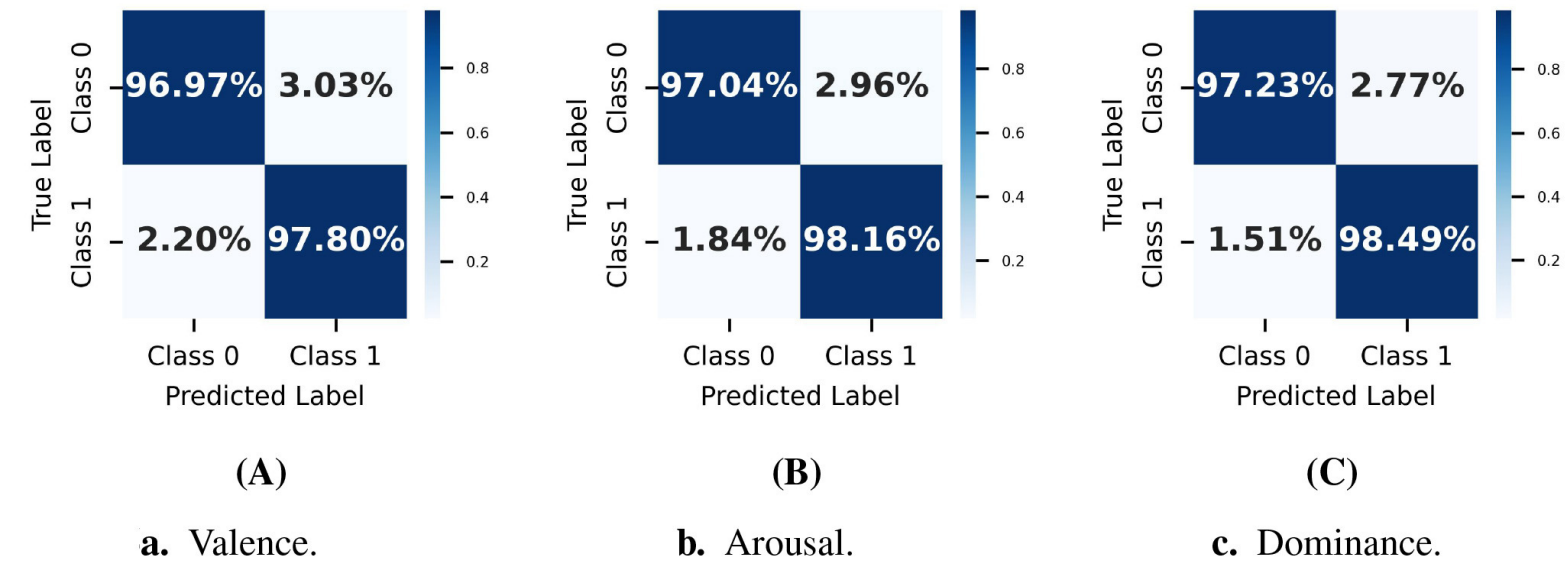
Confusion matrices on the DEAP dataset for (**A**) valence, (**B**) arousal, and (**C**) dominance dimensions.

**Figure 7:**
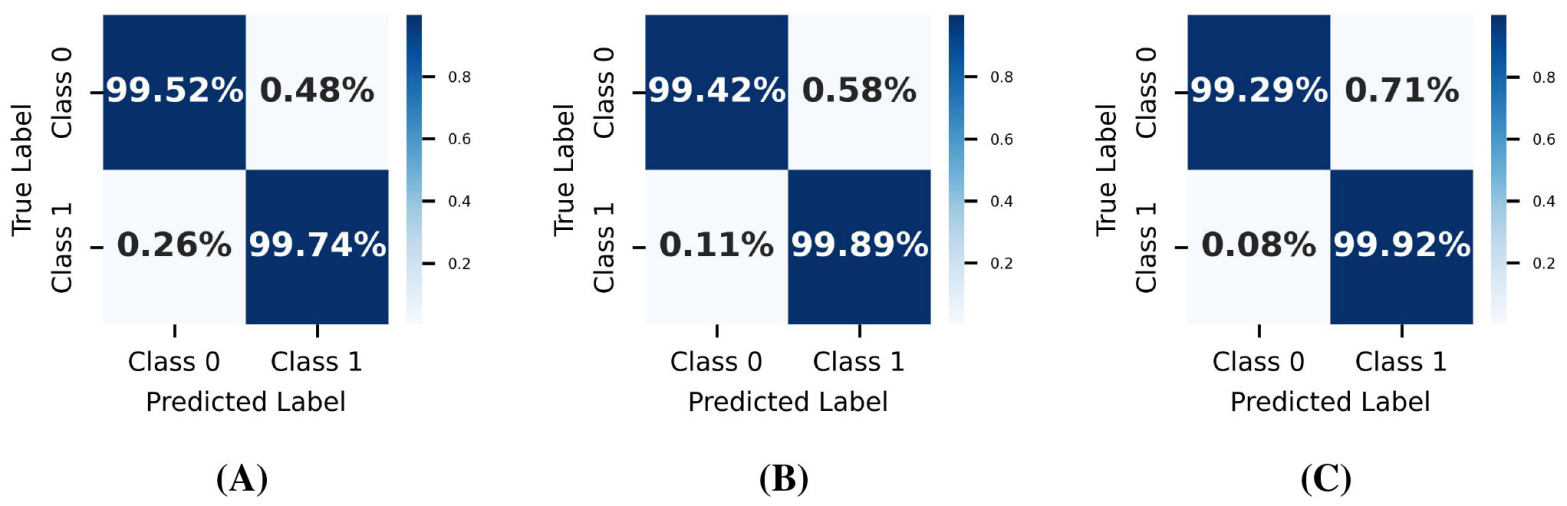
Confusion matrices on the DREAMER dataset for (**A**) valence, (**B**) arousal, and (**C**) dominance dimensions.

The DEAP and DREAMER datasets exhibit class imbalance in their binary classification labels, as detailed in Table 6, where low valence, arousal, and dominance are abbreviated as LV, LA, and LD, respectively, while high valence, arousal, and dominance are denoted as HV, HA, and HD. To provide a detailed evaluation of model performance under this condition, we conducted a visual analysis of the classification results using confusion matrices. In all presented results, Class 0 corresponds to low arousal/valence/dominance, while Class 1 represents high arousal/valence/dominance. Figures 6, 7 display the confusion matrices for the DEAP and DREAMER datasets in subject-dependent experiments, respectively.

**Table 6:** Number of positive and negative samples in the binary classification setting.

Dataset	HV	LV	HD	LD	HA	LA
DEAP	43,440	33,360	47,700	29,100	4,5240	31,560
DREAMER	52,210	33,534	68,517	17,227	65,808	19,936

The experimental results demonstrate that our proposed model delivers outstanding classification performance across all three emotional dimensions. As shown, the model achieved high classification accuracy for both labels. The diagonal elements of the confusion matrices are markedly more prominent than the off-diagonal elements, indicating that the model effectively differentiates between emotional states of different intensities and maintains a low rate of misclassification. Across all outcomes, the classification accuracy for Label 0 remains slightly lower than that for Label 1, yet still at an excellent level. These results validate the effectiveness and robustness of the proposed model in emotion recognition tasks.

### 4.2 Comparative analysis of attention strategies

To validate the advantages of our proposed brain region-constrained attention mechanism, we designed comparative experiments based on the Transformer architecture. We systematically evaluated five different attention configurations, including Brain Region-Constrained Attention with 15 fine-grained regions (BRCA-15), Brain Region-Constrained Attention with 5 coarse lobes (BRCA-5), standard global attention, random partition attention “local brain region + global brain” attention in EEG-based emotion classification tasks. All experiments were conducted on the DEAP dataset using subject-dependent 10-fold cross-validation, with performance assessed separately on valence and arousal dimensions.

**Figure 8:**
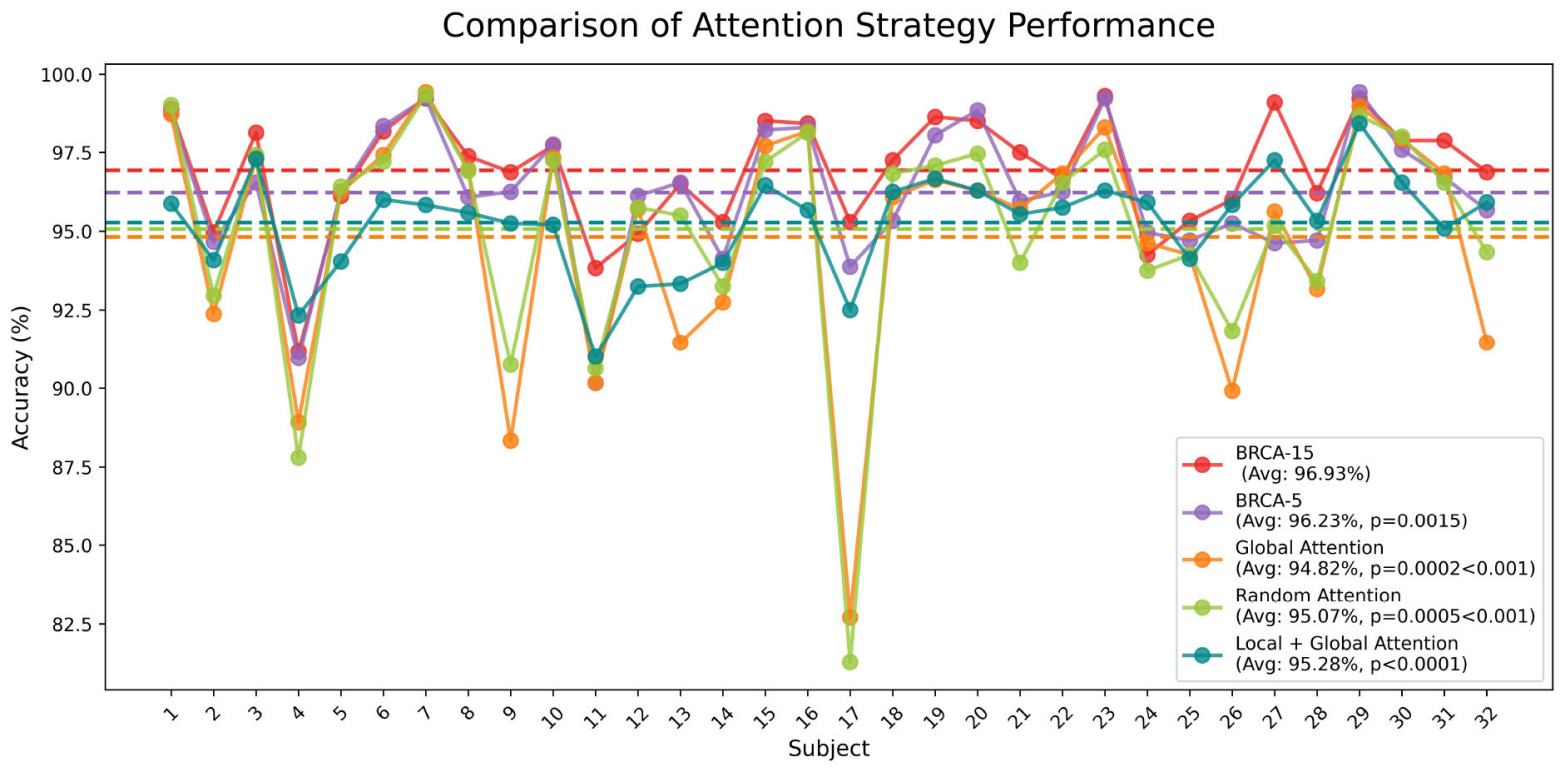
Comparison of classification accuracy across different attention strategies on the valence dimension (DEAP). The dashed line indicates the mean accuracy.

**Figure 9:**
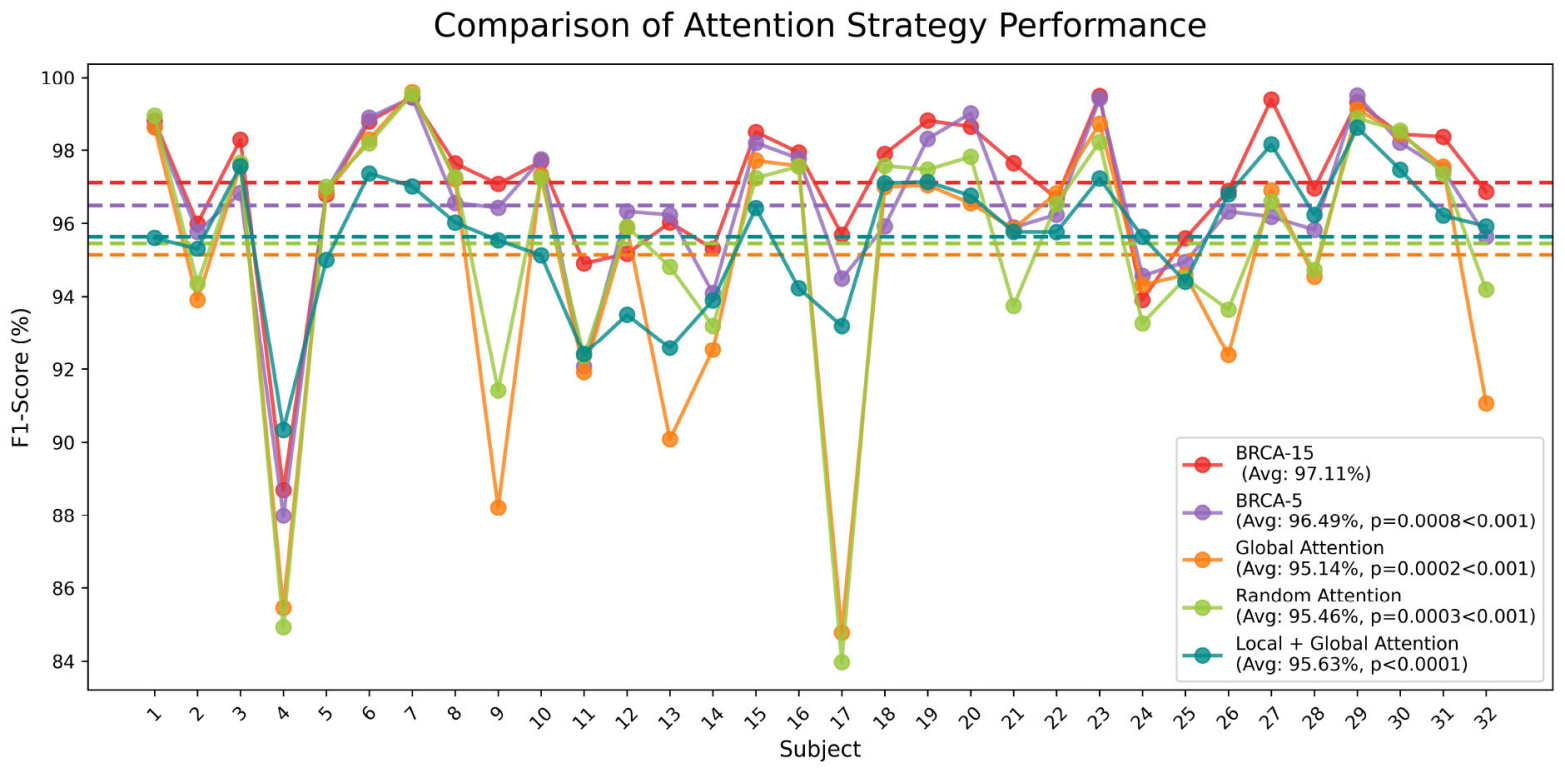
Comparison of classification F1-score across different attention strategies on the valence dimension (DEAP). The dashed line indicates the mean F1-score.

**Figure 10:**
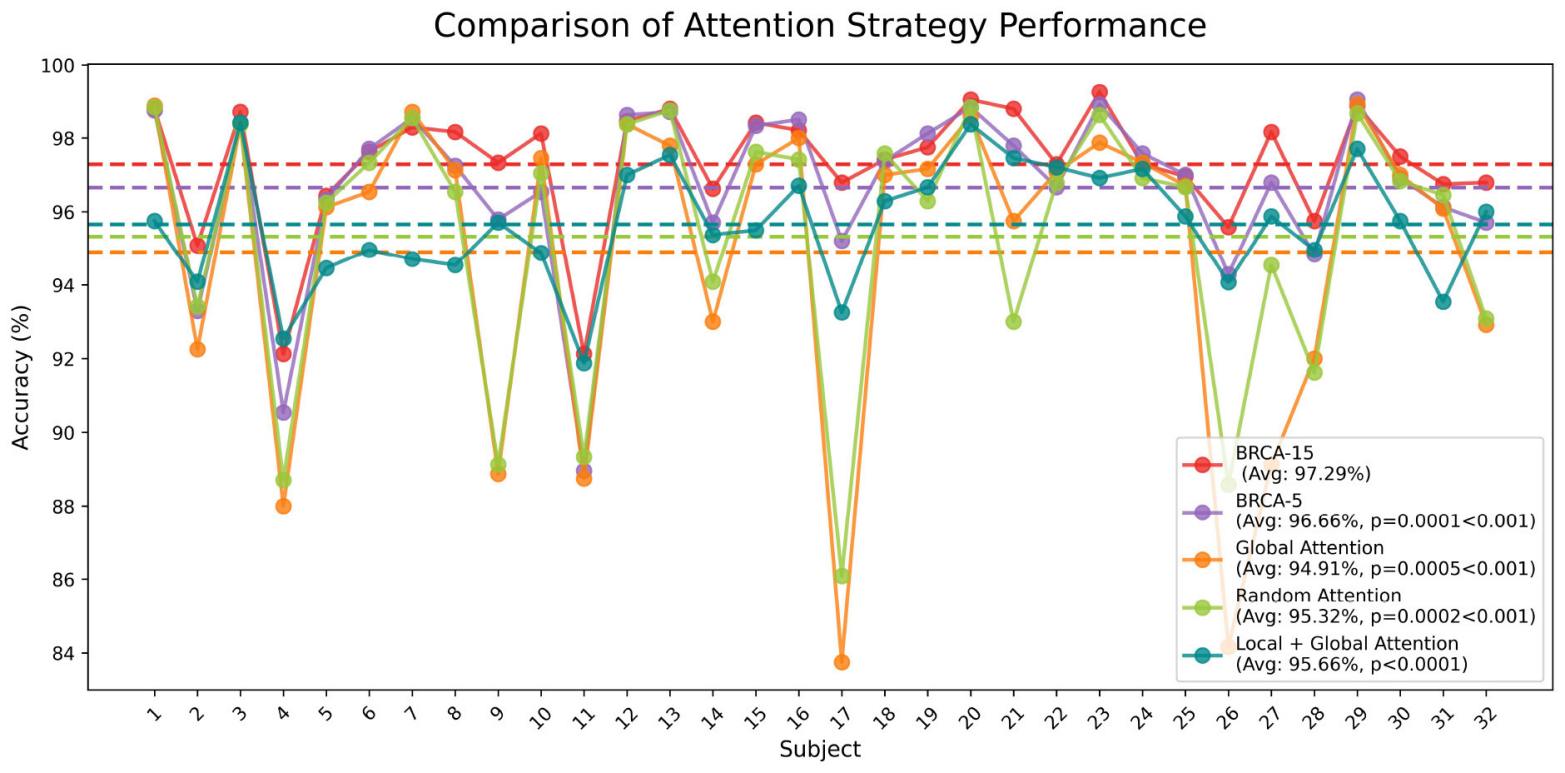
Comparison of classification accuracy across different attention strategies on the arousal dimension (DEAP). The dashed line indicates the mean accuracy.

**Figure 11:**
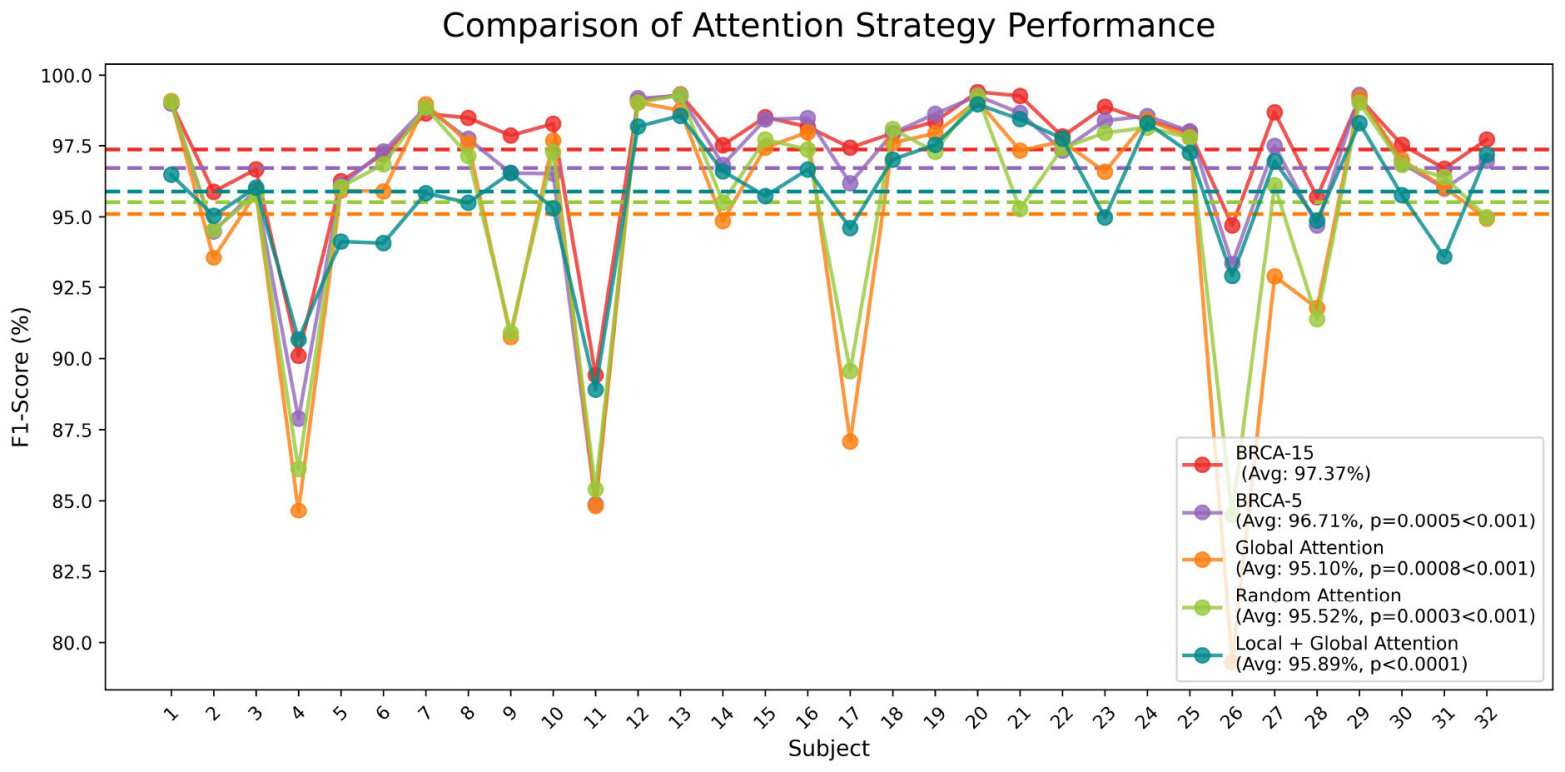
Comparison of classification F1-score across different attention strategies on the arousal dimension (DEAP). The dashed line indicates the mean F1-score.

The comparative results for valence dimension are presented in Figures 8, 9, while corresponding results for arousal dimension are shown in Figures 10, 11. In all figures, red, purple, orange, green and blue lines represent the performance of BRCA-15, BRCA-5, global, random partition and “local brain region + global brain” attention strategies, respectively, with corresponding dashed lines indicating their mean accuracy or F1-score values averaged across all subjects. The division scheme of the BRCA-5 brain regions is shown in Table 7.

**Table 7:** Electrode assignment of the BRCA-5 parcellation scheme.

Brain lobe	Electrode names
Frontal lobe	Fp1, AF3, F3, F7, Fp2, AF4, Fz, F4, F8, FC5, FC1, FC2, FC6
Central region	C3, Cz, C4
Parietal lobe	CP5, CP1, CP2, CP6, P7, P3, Pz, P4, P8
Temporal lobe	T7, T8
Occipital lobe	PO3, PO4, O1, Oz, O2

Considering the inherent class imbalance in emotion recognition datasets, we employed both accuracy and F1-score as complementary evaluation metrics to comprehensively assess different attention strategies. The F1-score, being the harmonic mean of precision and recall, provides particular value as it objectively evaluates the model's discriminative ability for both positive and negative emotion classes. This metric reflects not only the model's precision in identifying positive cases (e.g., high valence/arousal) but also its coverage of negative cases (e.g., low valence/arousal), thereby overcoming accuracy's limitations under class imbalance.

Experimental results demonstrate the superior performance of BRCA-15 across both emotional dimensions. For the valence dimension, brain region-constrained attention outperformed the global attention strategy with improvements of 2.11% in accuracy and 1.97% in F1-score. It also surpassed the random partition attention strategy, with gains of 1.61% in accuracy and 1.59% in F1-score. Compared to the “local brain region + global brain” attention strategy, our method achieved improvements of 1.65% in accuracy and 1.48% in F1-score, respectively. For the arousal dimension, the performance advantages were more substantial. Compared to the global attention strategy, accuracy and F1-score improved by 2.38% and 2.27%, respectively. Compared to the random partition attention strategy, the improvements were 1.97% and 1.85%, respectively. Furthermore, when evaluated against the “local brain region + global brain” attention, our method achieved a improvement of 1.63% in accuracy and 1.48% in F1-score. These consistent gains confirm that neuroscientific priors effectively guide the model to compute more discriminative channel weights, leading to enhanced feature representation and superior classification outcomes. This validates the dual value of integrating neuroscientific knowledge in improving both model interpretability and performance.

To further validate the statistical significance of our findings, we conducted paired *t*-tests on the results of the three attention strategies across 32 subjects, he detailed results are shown in Table 8. The results demonstrate that, for both the valence and arousal dimensions, the differences between the brain region-constrained attention and the other three attention strategies are highly statistically significant. These results confirm that the performance advantage of the brain region-constrained attention is not a chance occurrence, but rather consistent and robust. BRCA-5 also outperformed the three attention strategies without brain region constraints in both accuracy and F1-score across the valence and arousal dimensions. However, compared to BRCA-15, BRCA-5 showed decreases in both accuracy and F1-score, with the differences in accuracy on the arousal dimension and in F1-score on both dimensions reaching statistical significance (for accuracy on the valence dimension, the difference yielded a *p*-value of 0.0015).

**Table 8:** Statistical significance test results of attention strategies across different dimensions and metrics.

Attention strategy	Valence		Arousal	
	Acc	F1	Acc	F1
Global attention	*p* < 0.001	*p* < 0.001	*p* < 0.001	*p* < 0.001
Random attention	*p* < 0.001	*p* < 0.001	*p* < 0.001	*p* < 0.001
Local + global attention	*p* < 0.0001	*p* < 0.0001	*p* < 0.0001	*p* < 0.0001

Conventional global attention mechanisms treat EEG electrode channels as a one-dimensional sequence input and, when computing attention, implicitly assume that interactions between all channel pairs are physiologically meaningful. However, this assumption deviates significantly from neuroanatomical facts. Direct strong coupling between different brain regions is often lacking. Allowing a model to perform undifferentiated attention computation across all channels not only introduces a large amount of redundant cross-region information but may also lead the model to learn neurophysiologically implausible spurious correlations, thereby interfering with the identification of genuine emotion-related patterns.

The “local brain region + global brain” hybrid attention strategy attempts to model both intra- and inter-region information interactions. Its design retains cross-region attention computation, assuming that cross-region information interactions positively contribute to emotion recognition. However, based on our experimental results, this strategy underperforms compared to our proposed brain region-constrained attention mechanism. We attribute this phenomenon to the functional differences among brain regions in human activities. Effective long-range connections between brain regions are often sparse, and their functional coupling strength is generally weaker than the local connections within brain regions. Consequently, cross-region attention computation may introduce noise that is irrelevant to the emotion recognition task. This noise may arise solely from statistical regularities in the data, rather than from the genuine neural patterns underlying emotional activity. Over-learning such information may lead to overfitting to the training data, resulting in performance degradation on the test set.

In contrast, the advantage of our proposed brain region-constrained attention mechanism stems from its high consistency with the organizational principles of brain function. This mechanism explicitly models the neurophysiological characteristic of “strong intra-regional coupling and weak inter-regional coupling.” With a more parsimonious attention computation design, it aligns the model's learning process more closely with the brain's actual information processing patterns. This strategy effectively avoids learning the redundant information present in the “local brain region + global brain” attention strategy, thereby reducing the risk of overfitting, enhancing model generalization, and improving overall performance.

Further analysis at the individual-subject level reveals that both the brain region-constrained attention and the “local brain region + global brain” attention benefit from the incorporation of neuroscientific priors, exhibiting superior stability. For a few individuals (e.g., Subject 12), global attention achieves performance comparable to or even slightly better than brain region-constrained attention. Nevertheless, the brain region-constrained attention not only achieves the highest average performance but also exhibits the smallest cross-subject performance variability. Specifically, for the valence dimension, the standard deviation of accuracy for brain region-constrained attention is 1.87%, significantly lower than the 3.78% for global attention and the 3.70% for random partition attention. Its F1-score standard deviation is 2.13%, also markedly lower than the 3.82% and 3.57% for global and random attention, respectively. For the arousal dimension, the F1-score standard deviation for brain region-constrained attention is 2.30%, compared to 4.87% for global attention and 4.07% for random attention. The standard deviation for accuracy is 1.71%, again substantially superior to the 4.33% for global attention and the 3.60% for random attention. These results demonstrate that attention mechanisms incorporating neuroscientific priors possess stronger robustness. We hypothesize that when EEG signals have a relatively high signal-to-noise ratio (SNR), unconstrained attention mechanisms can also learn effectively. However, under conditions of poorer signal quality or lower SNR, the brain region-constrained attention maintains stable performance, highlighting its significant advantage in complex real-world scenarios.

Notably, both BRCA-15 and BRCA-5 adopt the proposed brain region-constrained attention mechanism, and both achieve significantly better recognition performance than the three attention strategies, namely global attention, random partition attention, and the “local brain region + global brain” attention. These results further validate the effectiveness of the proposed brain region-constrained attention mechanism. Comparing BRCA-15 with BRCA-5, the accuracy of BRCA-5 decreased by 0.7% and 0.63% on the valence and arousal dimensions of the DEAP dataset, respectively, while the F1-score decreased by 0.62% and 0.66%. These relatively small changes indicate that the model exhibits robustness to physiologically plausible partitioning schemes, while finer-grained region partitioning helps capture more discriminative local neural patterns, thereby further improving recognition performance. This finding also explains why the fine-grained 15-region scheme was chosen as the default configuration in our subject-dependent experiments and subject-independent experiments.

### 4.3 Ablation studies

To validate the effectiveness of individual components in our model, we conducted additional ablation studies on the DEAP dataset. Experiments were performed under the subject-dependent paradigm, employing 10-fold cross-validation, and evaluated across the valence, arousal, and dominance dimensions. Several model variants were designed for comparative analysis, with their configurations summarized in Table 9. Model 1 serves as a pure temporal baseline, devoid of attentional mechanisms. Model 2 introduces the complete spatio-temporal processing branch, including the proposed brain region-constrained attention, to evaluate its standalone efficacy. Model 3 acts as a reference for the spectral-spatial branch, implemented with standard residual blocks. Finally, Model 4 represents the enhanced version of that branch, incorporating multi-scale residual connections to assess their added value.

**Table 9:** Architectural configurations of the model variants used in the ablation experiments.

Model	ST Net		SS Net	
	Tem-CNN	BRC-Trans.	Std-Block	MSR-Block
Model 1	✓			
Model 2	✓	✓		
Model 3			✓	
Model 4			✓	✓
**BRAD-Net**	✓	✓	✓	✓

In Table 9, the "✓" symbol denotes that the module is included. Tem-CNN, temporal CNN; BRC-Trans., BRC transformer. The bold values indicate our proposed full model.

A core design element is the multi-scale residual block in the spectral-spatial branch. To validate its effectiveness, we compared Model 3 and Model 4. The model equipped with multi-scale residual modules demonstrated markedly superior classification performance, indicating that these modules, with their heterogeneous kernel sizes, successfully achieve multi-scale feature extraction while maintaining a low parameter count. This design aids in preventing overfitting and facilitates the extraction of highly discriminative features.

Table 10 summarizes the results of the ablation experiments. A comparative performance analysis across different network architectures confirms the contribution of each component to the overall classification efficacy. By comparing Model 2, Model 4, and our full model BRAD-Net, which incorporates a parallel dual-branch structure, we validate the necessity of fusing spatio-temporal and spectral-spatial features. The dual-branch structure significantly outperformed the single-branch architectures in both accuracy and F1-score across all three dimensions: valence, arousal, and dominance. Specifically, compared to the single spectral-spatial branch (Model 4), BRAD-Net increased accuracy by 4.58%, 4.46%, and 4.31%, and F1-score by 4.17%, 4.10%, and 3.90%, respectively. Compared to the single spatio-temporal branch (Model 2), it improved accuracy by 0.75%, 0.64%, and 0.82%, and F1-score by 0.74%, 0.66%, and 0.83%, respectively. These results confirm that integrating complementary multi-domain features directly enhances classification performance.

**Table 10:** Results of the ablation study on the DEAP dataset using subject-dependent 10-fold cross-validation.

Model	Performance metrics (accuracy/F1-score)		
	Valence	Arousal	Dominance
Model 1	96.48/96.76	96.81/96.95	96.96/97.16
Model 2	96.69/96.91	97.06/97.16	97.15/97.35
Model 3	90.13/91.02	90.66/91.30	91.53/92.31
Model 4	92.86/93.48	93.24/93.72	93.66/94.28
**BRAD-Net**	**97.44/97.65**	**97.70/97.82**	**97.97/98.18**

The bold values indicate the best results.

Furthermore, the superior performance of Model 2 over Model 4 suggests a varying importance of different feature types. We attribute this performance disparity to two factors. First, the DEAP dataset contains 32 electrode channels. To preserve their spatial distribution on the scalp, we mapped them onto an 8 × 9 2D matrix. The spectral-spatial branch employs a CNN-based multi-scale residual network, which relies on sliding local convolutional kernels to extract features in the 2D space. When the input feature map is relatively sparse, the receptive fields of the convolutional kernels may contain a large number of zero values or noise, leading to insufficient extraction of valid information. In contrast, the spatio-temporal branch uses continuous temporal EEG signals as input, avoiding this limitation and achieving more robust feature extraction.

Second, the spatio-temporal branch adopts a Transformer architecture, which enables the integration of neuroscientific priors into the attention computation process through the introduction of a brain region-constrained attention mechanism. This mechanism effectively enhances the model's capacity to extract discriminative features from EEG signals. This is further validated by the comparative experiment between Model 1 and Model 2. Experimental results across all three emotional dimensions consistently demonstrate the positive impact of this enhanced Transformer module on the model's overall classification performance.

### 4.4 Feature vector visualization

**Figure 12:**
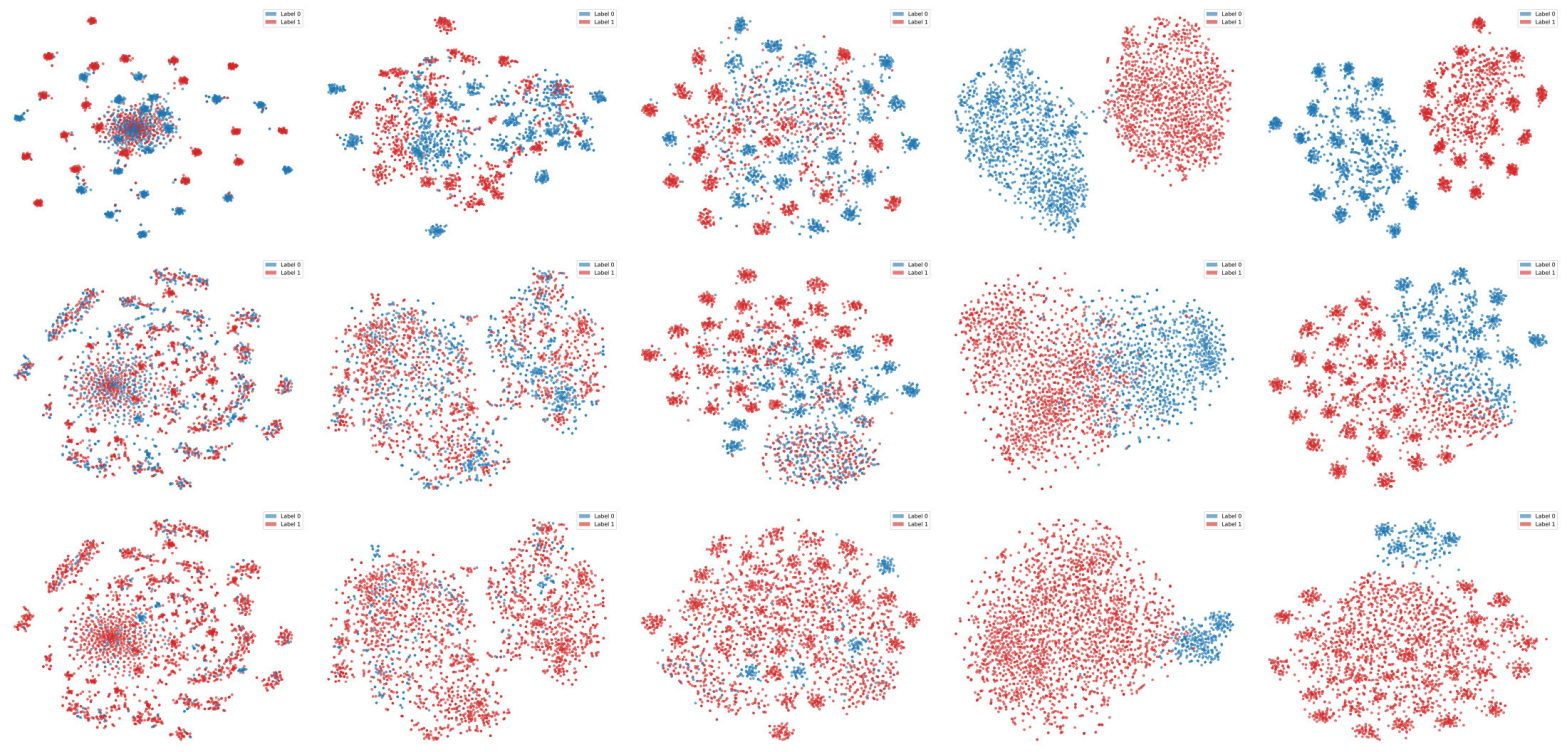
Feature vector visualization. Progressive visualization of learned EEG features (left to right): raw signals, combined DE and integrated PSD features, spatio-temporal features, spectral-spatial features, and final fused features.

To intuitively illustrate the evolution of features learned by our model at different levels, Figure 12 presents a column-wise visual comparison comparing the evolution of feature representations on the DEAP dataset, from raw input to the final fused output across different emotional dimensions. The first column displays three representative segments of raw time-series data. The second column shows the combined features derived from DE and integrated PSD. The third and fourth columns present the feature vectors extracted by the spatio-temporal branch and the spectral-spatial branch, respectively. The final column visualizes the output feature vectors after fusion through the dual-branch module. As demonstrated, from the raw signals to the single-branch features and further to the dual-branch fused features, both the intra-class compactness and inter-class separation improve markedly. The fused dual-branch features exhibit the clearest linearly separable pattern, with a decision boundary substantially more distinct than those of individual branch features. This visualization confirms that the proposed dual-branch architecture can effectively extract and hierarchically fuse complementary information from the spatio-temporal and spectral-spatial domains of the raw EEG signals, thereby learning more discriminative and classification-friendly robust feature representations.

### 4.5 Model parameter count and computational efficiency analysis

The proposed spatio-temporal and spectral-spatial dual-branch network strikes a careful balance between computational efficiency and recognition performance. The model employs lightweight multi-scale convolutional modules, replaces standard convolutions with depthwise separable convolutions, and adopts a streamlined brain region-constrained attention strategy to reduce the number of parameters and computational cost. To objectively evaluate the computational efficiency of the proposed model, we conducted comparative analyses of parameter count and computational cost on the DEAP and DREAMER datasets, selecting various multi-dimensional feature extraction models and comparison methods that adopt the “within-brain-region + cross-brain-region” interaction strategy. Table 11 presents the parameter count and floating-point operations (FLOPs) per sample for each model.

**Table 11:** Comparison of parameter count and computational cost (FLOPs) of different models on the DEAP and DREAMER datasets.

Model	DEAP		DREAMER	
	Params	FLOPs	Params	FLOPs
PconvCapsNet	2.880M	99.650M	-	-
MTSL	3.195M	-	-	-
MLF-CapsNet	110.850M	-	44.150M	-
FP-CapsNet	-	5020.000M	-	766.000M
LResCapsule	4.150M	295.960M	-	-
STRFLNet	-	-	25.150MB	-
Priyasad	7.5M		7.5M	
**BRAD-Net**	0.231M	483.569M	0.228M	259.528M

M denotes million (i.e., × 10^6^). “-” indicates that the corresponding result was not reported. The bold values indicate our proposed model.

As shown in Table 11, the proposed BRAD-Net model achieves a parameter count of only 0.231M on DEAP and 0.228M on DREAMER, which is the lowest among all comparison methods and significantly outperforms other models. In terms of computational complexity, the proposed model achieves FLOPs of 483.569M and 259.528M on the DEAP and DREAMER datasets, respectively, accounting for only 9.6% (DEAP) and 33.9% (DREAMER) of those of FP-CapsNet. This demonstrates that the proposed model achieves comparable or even superior recognition performance compared to these high-complexity models while maintaining low computational overhead. Overall, the proposed model achieves optimal or suboptimal performance in terms of both parameter count and computational cost, while maintaining competitive recognition accuracy. This combination of low parameter count, low computational cost, and high recognition accuracy makes our model particularly suitable for real-time EEG-based emotion recognition applications in resource-constrained scenarios, such as wearable brain-computer interface devices and mobile emotion monitoring systems. Furthermore, the lightweight model design implies faster inference speed and lower energy consumption, laying a solid foundation for practical deployment of the model.

### 4.6 Limitations and future work

The proposed BRAD-Net model performs well in EEG-based emotion recognition tasks, demonstrating competitive performance particularly in subject-dependent experiments. However, the results of subject-independent experiments indicate that there remains substantial room for improvement in the model's cross-subject generalization capability. Due to the inherent individual variability of EEG signals, the model struggles to learn a unified signal representation across different subjects, which is also a common challenge faced by the current field of EEG-based emotion recognition. In particular, when evaluating on the valence dimension of the DEAP dataset in cross-subject settings, BRAD-Net achieves an accuracy of 63.36%, which is lower than the 74.01% of PconvCapsNet. We attribute this performance gap to three main factors.

First, the difference in the number of channels between datasets. The DEAP dataset contains 32 electrode channels, substantially more than the 14 channels in the DREAMER dataset. A larger number of electrode channels enables the capture of richer spatial information. However, these fine-grained spatial features also amplify inter-individual signal distribution differences. In cross-subject transfer tasks, a larger number of channels entails more complex neural pattern representations and amplifies inter-individual distributional differences, thereby increasing the difficulty of cross-subject pattern recognition.

Second, the boundary misalignment issue of fine-grained brain region partitioning in cross-subject transfer. BRAD-Net adopts a fine-grained partitioning scheme consisting of 15 brain regions. This design achieves excellent performance in subject-dependent experiments, demonstrating that fine-grained partitioning helps capture more discriminative local features. However, in cross-subject transfer tasks, there exist natural differences in physiological structures across individuals (e.g., head shape, functional boundaries of brain regions), and the placement of the electrode cap may also be subject to slight physical shifts during data acquisition. These factors collectively lead to boundary shifts or misalignment across different individuals. This issue may be particularly pronounced in the DEAP dataset, which has a larger number of electrode channels and higher spatial resolution. More electrodes imply finer spatial partitioning and consequently a higher likelihood and greater impact of boundary misalignment. This partially explains why the cross-subject performance of BRAD-Net on the DEAP dataset is lower than that on the DREAMER dataset.

Third, the difference in model design philosophy. Our model adopts a brain region-constrained attention mechanism that strictly restricts attention computation to within-brain regions and completely prohibits cross-region information interaction. While this design achieves excellent performance in subject-dependent experiments, it may, in cross-subject generalization scenarios, lose some long-range neural patterns that are stable across individuals and valuable for generalization. In contrast, models such as PconvCapsNet, which allow cross-region information interaction, may have an advantage in cross-subject tasks by capturing such robust cross-region features.

To address this limitation, future work will focus on the following three directions. First, we will adopt adversarial domain adaptation or regularization methods to minimize the feature distribution discrepancy between the source and target domains, thereby enhancing the model's generalization capability in cross-subject scenarios. Second, we will design personalized fine-tuning strategies. Building upon a pre-trained model, we will develop fast and lightweight fine-tuning schemes for new users, rapidly adapting model parameters to fit individual users' EEG characteristics and mitigating the adverse impact of individual variability on model performance. Third, while preserving the core framework of brain region constraints, we consider introducing a lightweight low-rank cross-region attention leakage module that allows a small amount of learnable information flow between brain regions. This design retains the core advantages of the original brain region-constrained attention while providing a small number of learnable degrees of freedom to accommodate individual differences. In the context of domain shift during cross-subject transfer, this module enables the model to adaptively learn which cross-region connections are beneficial for generalization without compromising the structurally guided within-region attention. It provides the model with a certain degree of flexibility without significantly increasing computational overhead, alleviates the data drift issue caused by individual differences, and improves the model's performance in subject-independent experiments.

The current study relies solely on EEG signals for emotion recognition. Although EEG signals possess good discriminative capability for emotion recognition, emotion is a complex physiological process that cannot be fully characterized by a single type of physiological signal alone. Multiple physiological signals, such as electrocardiogram (ECG) and galvanic skin response (GSR), can provide complementary information related to emotional states. The proposed BRAD-Net is essentially a multi-source information synergistic fusion framework. Currently, we have achieved multi-domain feature synergistic modeling of EEG signals, a design that can be naturally extended to a multi-modal information synergistic framework. In future work, we plan to extend BRAD-Net to a multi-modal version, exploring the synergistic constraint mechanisms among multi-modal physiological signals, including EEG, ECG, GSR, and facial expressions. Through the complementary fusion of cross-modal information, we aim to further improve the accuracy, stability, and generalization capability of emotion recognition. We believe that this direction will not only address the limitation of incomplete single-modal information but also drive the model toward a more physiologically grounded, multi-modal perception paradigm.

Furthermore, the current brain region partitioning scheme in this paper is based on a standard anatomical atlas and adopts a rigid, fixed partitioning strategy. It does not fully account for unstable factors, such as individual differences in head shape and functional boundaries of brain regions, as well as slight shifts in electrode cap placement across different recording sessions. These factors may lead to misalignment between the predefined hard boundaries and the actual functional brain region distributions of individual subjects, thereby affecting the model's generalization performance in cross-subject scenarios. To address this limitation, future work will explore more adaptive dynamic brain region partitioning methods, such as subject-specific functional connectivity-based adaptive partitioning, or consider introducing learnable soft boundary adjustment mechanisms to enhance the model's robustness and generalization capability.

## 5 Conclusion

This paper proposed a novel BRAD-Net, which incorporates a brain region-constrained attention mechanism and a lightweight multi-scale residual network architecture. The model effectively addresses the insufficient utilization of multi-dimensional features in EEG-based emotion recognition. Unlike traditional global attention methods, the spatio-temporal branch of BRAD-Net implements brain region partitioning at the electrode level and employs a functionally constrained attention mask to suppress cross-region interference, which significantly enhanced feature discriminability. Furthermore, the dual-branch structure comprehensively captures both temporal dynamics and spectral-spatial information for emotion recognition. Experimental results demonstrate that our model outperforms other recent works under both subject-dependent (DEAP and DREAMER) and subject-independent (DREAMER) experimental paradigms. Furthermore, a complementary experiment on a depression dataset (see Appendix 1) further validates the powerful generalization capability of our model across different tasks.

Beyond its empirical contributions, this work underscores a significant methodological shift towards the deep integration of domain knowledge into computational architecture. Moving beyond purely data-driven paradigms, the proposed brain region-constrained attention mechanism exemplifies a collaborative, knowledge-guided approach, where established neuroscientific principles are embedded as structural priors within the learning algorithm itself. Notably, the design of restricting attention computation to within-brain regions provides a new theoretical perspective on the application of brain parcellation knowledge in classification models. Particularly, the design that leverages only within-brain-region information for attention computation provides a fresh theoretical insight into how brain parcellation knowledge can be applied in classification models. We contend that such a direction is vital for developing trustworthy and insightful computational models in affective computing and beyond.

## Figures and Tables

**Figure A1:**
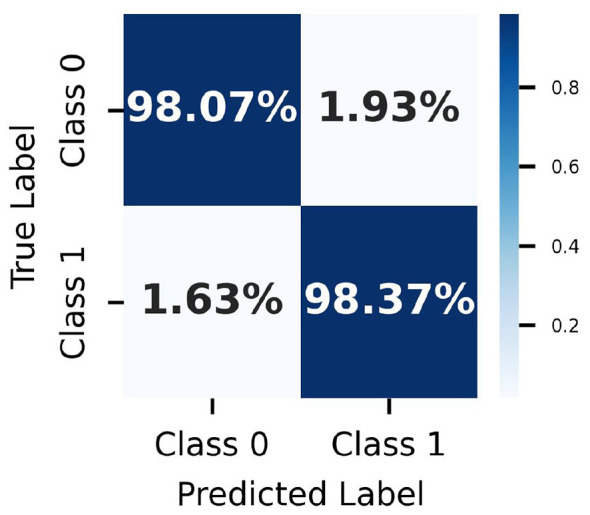
Confusion matrix for the depression classification task.

## Data Availability

Publicly available datasets were analyzed in this study. The DEAP dataset used in this study was introduced in https://doi.org/10.1109/T-AFFC.2011.15; the DREAMER dataset is available at https://zenodo.org/records/546113; and the depression detection dataset is available at https://figshare.com/articles/dataset/EEG_Data_New/4244171.

## Appendix

### Appendices: cross-paradigm validation of BRAD-Net for depression detection

To further assess the generalizability of the proposed method beyond emotion-specific paradigms, we evaluated its performance on the publicly available depression EEG dataset published by Mumtaz (2016). This dataset comprises resting-state EEG recordings from 34 patients diagnosed with major depressive disorder (MDD) and 30 healthy control (HC) subjects, collected under both eyes-open (EO) and eyes-closed (EC) conditions.

Using 10-fold cross-validation on the combined EO and EC data, we employed the average performance across all folds as the final metric. The proposed model achieved a high classification accuracy of 98.21% and an F1-score of 98.15%, demonstrating robust discriminative capability between depressive and healthy states. The corresponding confusion matrix is presented in Figure A1, where Class 0 denotes the depression group and Class 1 represents the healthy control group. The matrix reveals an extremely low misclassification rate across both classes, confirming the model's reliability alongside its high accuracy.

These results underscore two important aspects: first, the proposed framework demonstrates robust discriminative power in a clinically challenging binary classification task (MDD vs. HC), confirming its ability to capture neurophysiological signatures relevant to mental states; second, the model exhibits strong adaptability across distinct EEG experimental paradigms–from emotion-elicitation tasks to resting-state recordings–and generalizes effectively to diverse downstream classification objectives. This dual capability highlights the method's potential as a versatile and reliable tool for EEG-based biomarker discovery and computational psychiatry.
